# Dissociating representations of affect and motion in visual cortices

**DOI:** 10.3758/s13415-023-01115-2

**Published:** 2023-08-01

**Authors:** James H. Kryklywy, Brandon J. Forys, Joana B. Vieira, Derek J. Quinlan, Derek G. V. Mitchell

**Affiliations:** 1 Department of Psychology, Lakehead University, Thunder Bay, Canada; 2 Department of Psychology, University of British Columbia, Vancouver, Canada; 3 Department of Psychology, University of Exeter, Exeter, UK; 4 Department of Psychology, Huron University College, London, Canada; 5 Graduate Brain and Mind Institute, Brain and Mind Institute, University of Western Ontario, London, Ontario N6A 5B7 Canada; 6 Department of Anatomy & Cell Biology, University of Western Ontario, London, Canada; 7 Department of Psychology, University of Western Ontario, London, Canada; 8 Department of Psychiatry, University of Western Ontario, London, Canada

**Keywords:** Emotion, Motion perception, Threat response, Approach and avoid, fMRI, RSA, IAPS images

## Abstract

While a delicious dessert being presented to us may elicit strong feelings of happiness and excitement, the same treat falling slowly away can lead to sadness and disappointment. Our emotional response to the item depends on its visual motion direction. Despite this importance, it remains unclear whether (and how) cortical areas devoted to decoding motion direction represents or integrates emotion with perceived motion direction. Motion-selective visual area V5/MT+ sits, both functionally and anatomically, at the nexus of dorsal and ventral visual streams. These pathways, however, differ in how they are modulated by emotional cues. The current study was designed to disentangle how emotion and motion perception interact, as well as use emotion-dependent modulation of visual cortices to understand the relation of V5/MT+ to canonical processing streams. During functional magnetic resonance imaging (fMRI), approaching, receding, or static motion after-effects (MAEs) were induced on stationary positive, negative, and neutral stimuli. An independent localizer scan was conducted to identify the visual-motion area V5/MT+. Through univariate and multivariate analyses, we demonstrated that emotion representations in V5/MT+ share a more similar response profile to that observed in ventral visual than dorsal, visual structures. Specifically, V5/MT+ and ventral structures were sensitive to the emotional content of visual stimuli, whereas dorsal visual structures were not. Overall, this work highlights the critical role of V5/MT+ in the representation and processing of visually acquired emotional content. It further suggests a role for this region in utilizing affectively salient visual information to augment motion perception of biologically relevant stimuli.

**Supplementary Information:**

The online version contains supplementary material available at 10.3758/s13415-023-01115-2.

Imagine reaching out to hand a piece of cake to an eagerly awaiting child. Now imagine pulling that cake away before the child is able to taste it. The emotional response to the cake shifts; excitement becomes disappointment. The response to the dessert depends on the motion. While the cake itself does not change, its motion towards or away from the child both can create two immediate and potent, yet diametrically opposed, reactions. Motion information is meaningful in how we appraise the world and what behaviours may need to be selected to respond appropriately. Determining whether, and how, areas of the brain involved in motion processing can integrate emotional valence is needed for a comprehensive understanding of how an organism updates their perceptional experience to reflect the behavioural significance of objects around them.

When visual information reaches occipital areas, it diverges along canonical dorsal and ventral pathways (Milner and Goodale, 1993); dorsal visual regions mediate the execution of visually guided actions towards objects in our environment, while ventral visual regions mediate our perception of the visual world (an "action" stream vs. "perception" stream for visual processing; Milner and Goodale, 2008). Distinct functional differences exist between these two pathways (the precise nature of which is still under investigation; see Brogaard, 2012; Foley et al., 2015; Freud et al., 2016; Mann et al., 2021), yet they maintain extensive bilateral communication (Buchel and Friston, 1997; Milner and Goodale, 2008; Kravitz et al., 2013).

Emotionally relevant objects typically enhance processing in visual cortices (Vuilleumier, 2005; Pessoa and Adolphs, 2010). Yet evidence of this effect is observed mainly with processes mediated by the ventral-stream, including object identification and perceptual quality (Mitchell and Greening, 2012). While enhancing these perceptual features of biologically significant stimuli is likely adaptive, other characteristics may be equally important. For example, accurately representing the position and movement of threatening objects or determining whether the approaching object is harmful or desired are both critical tasks in response selection. (Panksepp, 1990; Panksepp, 1998; Mobbs et al., 2009; Aupperle et al., 2015; Qi et al., 2018; Meyer et al., 2019; Mobbs et al., 2020; Yeung and Chan, 2020). Consistent with the importance of affect in behavioural guidance, emotional valence has been shown to modulate activity in motion-processing areas in the human brain, including area V5/MT+ (Attar et al., 2010; Kolesar et al., 2017).

The visual processing structure V5/MT+ often is defined by its general motion-sensitivity (Zeki et al., 1991; Tootell et al., 1995b; Huk et al., 2002), making it a prime target for investigation of motion-emotion interactions. V5/MT+, however, is not a homogenous region; it contains at least four subregions, each characterized by specific organizational patterns and reactivity (e.g., retinotopic wedges or ipsi vs. contralateral motion; Kolster et al., 2010; Gao et al., 2020). Although human neuroimaging studies often are unable to examine the V5/MT+ complex with enough spatial resolution to detect *all* of these subregions independently, distinct zones sensitive to global flow versus general motion often are observed (Smith et al., 2006; Ohlendorf et al., 2008; Gaglianese et al., 2023).

Definitive placement of V5/MT+ complex in dual pathway frameworks of visual processing is a challenge (Born and Bradley, 2005; Gilaie-Dotan et al., 2013; Kravitz et al., 2013). While traditionally associated with the dorsal visual stream (Born and Bradley, 2005; Arall et al., 2012; Cloutman, 2013; but see Gilaie-Dotan, 2016), V5/MT+ also displays patterns of connectivity consistent with ventral stream structures (Nassi and Callaway, 2007; Abe et al., 2018). For example, both V5/MT+ and ventral visual regions—but not dorsal visual regions—display robust anatomical and functional connectivity with the amygdala (Baizer et al., 1993; Young et al., 1994; Amaral et al., 2003; Furl et al., 2013)—a region often noted for its role in emotional processing (LeDoux, 1992; Vuilleumier, 2005). Further commonalities between processing in V5/MT+ and ventral visual structures include their susceptibility to optical illusions (Tootell et al., 1995a; He et al., 1998; Antal et al., 2004) and their sensitivity to emotion in postures (de Gelder and Hadjikhani, 2006) and dynamic facial expressions (Furl et al., 2013). This contrasts with patterns observed for dorsal stream processes, which appear less sensitive to optical illusions (Aglioti et al., 1995; Haffenden and Goodale, 1998) and where the impact of emotion is less clear. For example, observation of emotion-based increases in neural activity in dorsal visual areas (de Gelder et al., 2004; Goldberg et al., 2012, 2014; El Zein et al., 2015; Engelen et al., 2015; Solanas et al., 2020) are contrasted by null effects of emotionally salient environments on dorsally mediated behaviours (e.g., visually guided targetting; Kryklywy and Mitchell, 2014; Enns et al., 2017).

Extending canonical classification of visual pathways, a third visual processing stream—one dedicated to social perception—has been recently proposed (Pitcher and Ungerleider, 2021). Notably, many of the motion-sensitive “dorsal” structures identified in previous work as processing emotional information, such as those sensitive to social response planning (Kong et al., 2021) and body posture (de Gelder et al., 2015; Engelen et al., 2015; Zhan et al., 2018), also are implicated in this newly outlined processing stream. These include the V5/MT+ complex and the posterior superior temporal sulcus (de Gelder et al., 2004; Goldberg et al., 2014; El Zein et al., 2015; Engelen et al., 2015). Furthermore, given the extensive cross stream connectivity throughout visual processing areas, it remains unclear whether emotional information is independently decoded in V5/MT+ and other motion sensitive regions, or whether emotion representation in these structures is solely the result of feedforward signalling from other visual processing areas.

We used functional magnetic resonance imaging to investigate the interaction between emotion and visual motion perception by leveraging the motion aftereffect (MAE; Tootell et al., 1995a). Specifically, we deployed a two-pronged approach to determine the impact of perceived motion direction and emotional content on neural processing in motion sensitive regions. We first used univariate statistical analyses to target emotional reactivity in functionally localized regions of interest (ROIs) sensitive to stimulus motion (with a particular emphasis on V5/MT+). This was followed by a multivariate statistical approach to determine how representation of emotional information varies across visual processing regions. Specifically, these analyses interrogate whether representations of emotional information is shared, i.e., similar in content and strength information, or unique between regions; the former indicated information likely propagated between structures, and the later indicative of emotional processing in the region. Participants viewed concentric rings that approached (expanded), receded (contracted), or alternated motion direction, followed by a static target image with positive, negative, or neutral emotional content. Based on the MAE (Wohlgemuth, 1911; Mather et al., 1998), static images presented after coherent motion appear to move in the opposite direction (to recede following approaching motion or to approach following receding motion) while images that appear after alternating motion patterns should appear static. To determine what type of information influences patterns of neural activation in response to the emotional events, pattern component modelling (PCM; Kriegeskorte and Kievit, 2013; Diedrichsen et al., 2018; Kryklywy et al., 2023) was conducted to estimate response-pattern similarities in different brain regions and to determine the extent to which these were driven by specific representational patterns of interest (POIs, e.g., presented visual motion, perceived visual emotion, emotional experience, etc.). These multivariate analyses were conducted in concert with more traditional univariate approaches. Specific ROIs were primary visual cortex (V1), ventral visual structures (vVS), dorsal visual structures (dVS), and visual area V5/MT+. These structures were targeted to investigate the extent of emotional representations in areas assigned to particular visual processing streams, and particularly for V5/MT+, wherein the designation within these streams is unclear.

From previous research describing anatomical connectivity between neural regions processing visual motion and emotion (Amaral and Price, 1984; Young et al., 1994; Amaral et al., 2003) and the behavioural effects observed in actions recruiting ventral visual regions (Lang et al., 1998; Kryklywy and Mitchell, 2014), we predicted that both the strength of the perceived MAE and its neural correlates should be influenced by emotional valence and motion direction. Behaviourally, we expected images that appeared to be approaching would elicit higher emotional arousal ratings similar to that observed during actually looming stimuli (Mobbs et al., 2010; Coker-Appiah et al., 2013; Low et al., 2015) as well as to illusions of motion and spatial distance (Muhlberger et al., 2008). Neurally, we predicted that both individually and group defined V5/MT+ ROIs would display increased activation for emotional compared to neutral images, similar to those previously observed in ventral visual areas (Morris et al., 1998; Vuilleumier and Driver, 2007). Secondary to these predictions, emotion was not expected to modulate activity in regions canonically associated with the dorsal visual stream but was expected to impact activity throughout the ventral visual pathway. For the multivariate analysis, we predicted that in visual structures, we would identify representational pattern components consistent with their canonical roles: V1 representations would include representational patterns for multiple low-level features. We predict that while ventral visual structures would maintain these representations, dorsal visual structures would not represent emotion- or identity-based features (Kryklywy et al., 2013; 2018; Kryklywy and Mitchell, 2014). Consistent with behavioural evidence suggesting that area V5/MT+ shares overlapping anatomical and functional architecture with ventral visual structures (Baizer et al., 1993; Young et al., 1994; Tootell et al., 1995a; He et al., 1998; Amaral et al., 2003; Antal et al., 2004; Furl et al., 2013), it was expected to represent both motion and emotion components.

## Methods

### Subjects

Nineteen healthy human subjects (13 females; 6 males; x̄_age_ = 22.6, standard deviation [SD] = 3.70) participated in the experiment. All participants reported no history of neurological or psychiatric illness, were right-handed, had normal hearing, normal or corrected-to-normal vision, and were fluent English speakers. This sample size provides the ability to detect medium-to-large effect sizes in our primary analyses (3 x 3 repeated-measures ANOVAs; see below) greater than 0.35 (Sullivan and Feinn, 2012), with a critical F of 2.66 at a power of 0.80. This effect size consistent with those calculated from previous neuroimaging work investigating motion aftereffects in the V5/MT+ complex (Taylor et al., 2000; Huk et al., 2001), adjusted for potential inflation related to post-hoc calculations (Reddan et al., 2017; Funder and Ozer, 2019). The study was approved by the Health Science Research Ethics Board at the University of Western Ontario.

### Stimuli and apparatus

Four unique motion video clips were created for this study in VPixx (VPixx Technologies). Two unique video clips were utilized as adaptation stimuli to induce the motion after-effect in the main experimental task. These consisted of concentric circles (1.64 cycles/degree; alternating black/white), which contracted or expanded relative to a central fixation point at a constant rate of 2 Hz (1.2 degrees/s). After-effects created by these stimuli are a perceptual drift of images in the opposite direction of the initial movement (Bowditch and Hall, 1882). A third video, consisting of concentric circles that alternated between expansion and contraction (motion at 2 Hz/1.2 degrees/s) every 2 seconds, was used as a no-after-effect control condition. A fourth video was created for the V5/MT+ localizer consisting of 200 short line segments (0.5º visual angle) randomly oriented either vertically or horizontally and moving at a constant velocity in one of eight directions for 20 s (3.28 º/s).

Twenty-seven images were chosen from the *International Affective Pictures System* stimulus set (IAPS: Lang et al., 2008; Supplemental Table ST1). To aid in the selection of these images, a pilot study (n = 10) was conducted to ensure that the spatial layout of visually salient features did not vary between emotional categories. For this pilot study, participants were presented with 78 IAPS images (35 negative, 25 neutral, and 35 positive). Image valence was defined based on the standardized rating manual provided with the IAPS stimulus set, which included 9-point Likert scale ratings for valence and arousal (higher scores indicated greater pleasantness and arousal respectively; image rating was conducted on a 9-point Likert scale; Lang et al., 2008). Participants were presented each stimulus for 2 s and were instructed to freely view each image during this time. Eye-behaviour was monitored using a fast video-based eye-tracker at 1,000 Hz (EyeLink 1000, SR Research). Subsequent analysis examined the percentage of time spent fixated within the centre of the image (<20° from the midpoint) in contrast to the periphery (>60° from the midpoint) for each image (reported as %C - %P). Nine pictures were chosen from each emotional category that allowed for the best matched spatial salience (i.e., regions of the visual field that held gaze during free viewing) between categories (positive vs. neutral: t_(16)_ = 0.12 *p* > 0.10; negative v. neutral: t_(16)_ = 0.15; *p* > 0.10; positive v. negative: t_(16)_ = 0.29; *p* > 0.10). Following this, additional analyses were conducted using these standardized ratings of the stimuli to ensure that the resulting positive and negative stimuli were balanced for arousal (positive = 5.40, negative = 5.52; t_(16)_ = 0.47; *p* > 0.10) and absolute valence deviations from neutral (positive = 7.56, negative = 2.86, scale-defined neutral = 5; t_(16)_ = 1.52; *p* > 0.10). In addition, physical properties were compared between categories to ensure a match on low-level visual features by subjecting each image to a wavelet analysis similar to that used previously (Delplanque et al., 2007). For this, each red, green, blue, and grayscale layer of the image was decomposed into eight frequency bands (512, 256, 128, 64, 32, 16, 8, 4, and <2 cycles per image). An examination of the energy within each band indicated no significant differences between the positive, negative, or neutral stimulus categories (all corrected *ps >* 0.10). Furthermore, absolute luminance levels did not significantly differ between categories (positive vs. neutral: t_(16)_ = 0.069; *p* > 0.10; negative vs. neutral: t_(16)_ = 0.89; *p* > 0.10; positive vs. negative: t_(16)_ = 0.93; *p* > 0.10). Due to the constraints imposed by equating the objective visual properties of the images across categories, other content-related visual features were unable to be matched. For example, of the nine stimulus exemplars in each of the positive, negative, and neutral image sets, five, two, and zero images contain human features respectively (see ST1 for full stimulus index). In addition, eight different neutral IAPS images were selected for use in a prescan practice task.

All stimuli were presented in the scanner by using a Silent Vision™ Extended Range XR Fiber Optic Visual System (SV-7021), allowing for presentation of stimuli within a 30° (horizontal) by 23° (vertical) field of view, which was filled by both the adaptation and test stimuli. A prescan practice task consisting of a shortened version of the motion-adaptation paradigm with MAEs induces on nonemotional target stimuli (see Supplemental materials for additional details) was conducted on a Lenovo ThinkPad W540 (1,920 X 1,080 pixels, refresh rate 60 Hz) at a viewing distance of ~40 cm.

### Procedure

#### V5/MT+ localization

Before the anatomical scan and experimental task, participants completed a single functional localizer run to identify area V5/MT+ by using conventional methods (Tootell et al., 1995b; Huk et al., 2002; Emmerling et al., 2016). The run began with an initial 3-s fixation period. Following this, participants were presented with moving line segments (18 s), a fixation cross on an otherwise blank screen (1-3 s), stationary line segments (18 s), and an additional fixation cross (1-3 s; 40 s per cycle total). This sequence was repeated 8 times within a run followed by a 12-s fixation period, for a total run duration of ~5 min, 35 s. Of note, the localizer paradigm utilized visual motion in multiple directions for each motion trial as opposed to the standard contract/expanding motion patterns to ensure that there would be no inadvertent MAEs created.

#### Experimental task

**Fig. 1:**
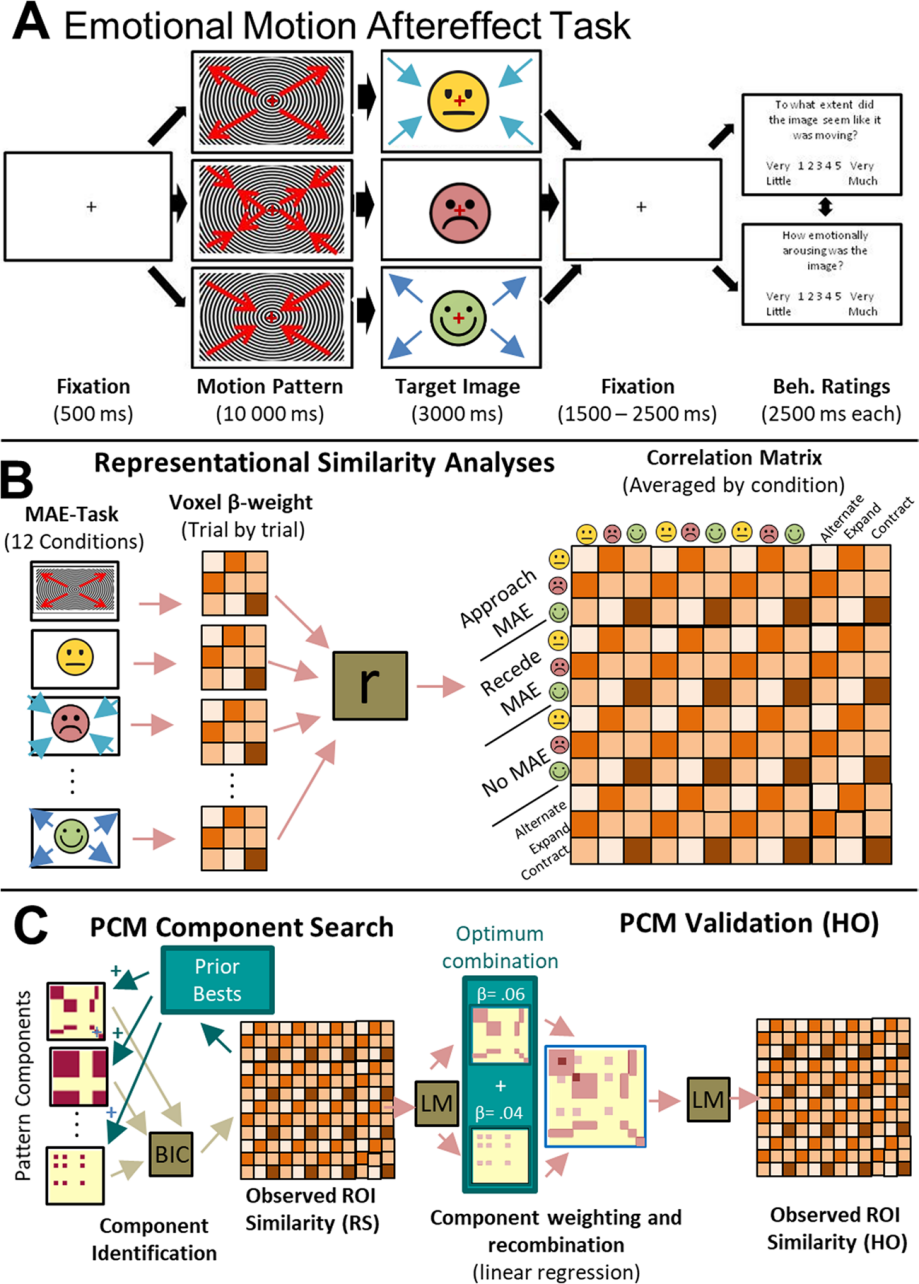
MAE paradigm and RSA-PCM schematics. (**A**) In the emotional MAE task, following a brief fixation period, participants were presented with a pattern of consistent motion that was expanding from a central fixation point (producing a receding MAE), contracting to fixation (producing an approaching MAE), or alternating direction between expansion and contraction around fixation (producing no MAE). Direction of motion in the adaptation videos and MAEs are indicated by red and blue arrows respectively and appear in the figure only for illustrative purposes. Emotion icons indicate potentially emotionality of complex scenes and are not representative of the target stimuli used. Following each trial, two questions were presented in random order to assess the impact of direction and emotion on the perception of illusory motion and emotional arousal. (**B**) Representational similarity analyses were conducted comparing voxel-wise similarity of conditions across all individual experimental runs and task component (adaptation, aftereffect, and motion conditions). These were subsequently averaged across run resulting in a similarity matrix indicating representational similarity across, and within conditions. (**C**) Pattern component analyses were performed to determine component patterns of the observed representational states for each ROI. Predefined components were fit to the representational patterns of the random sample (RS) using Bayesian information criterion (BIC) analyses. The best fitting component was held in the model (green box/arrow) and fit with the observed data in combination with all remaining components independently. This process was repeated until the optimum component combination was identified. A linear regression model (LM) was used to fit the identified components to the RS similarity data. A new reconstructed component was then created by combining the weighted RS component models and fit to the held-out participants (HO)

In the scanner, participants performed the “emotional MAE task” (Fig. 1A). To acclimatize to task timing, participants completed a short practice version of the scanning task before entering the scanner. Individual runs began with an initial fixation period of 2.5 s and ended with a fixation period of 14 s. Each trial within a run began with a 0.5-s static fixation cross, followed by a motion video (10 s; contracting, expanding or alternating). Immediately after the cessation of this video, one of 27 target images was presented for 3 s. During the presentation of both the motion video and the target image, participants were instructed to maintain fixation on a centrally located red fixation cross. Pending the motion video that had preceded them, perception of the target images was subject to visual motion-aftereffect, wherein they appeared to be moving in the opposite direction of the preceding stimulus. The utilization of MAEs allows for explicit control of the visual properties of the imagery across motion directions such that the perceived motion direction can be manipulated while keeping the actual physical parameters of the affective picture unchanged. Spatial frequencies and spectral components of the images remained stable within emotion conditions, yet the motion properties varied. After presentation of the target image, a black fixation cross appeared for 1.5, 2.0, or 2.5 s (randomized between trials) followed by two questions in random order: “To what extent did the image move [towards you/away from you/ around]?” (illusion quality rating) and “How emotionally arousing was the image?” (emotional arousal rating). All responses were given on a five-point Likert scale ranging from “Very Little” to “Very Much” and collected with an MRI-compatible five-response button box. Each question was presented for 2.5 s. A single run consisted of an image from each emotional category (positive, negative, neutral) presented following each of the motion patterns (contracting, expanding, alternating) three times, resulting in 27 trials per run (with individual run duration ~9 m 30 s). Trial orders were randomized within each run for each participant. Participants completed six experimental runs (162 total trials = 18 trials per combination of 3 motion adaptation directions x 3 test-stimulus emotional valences, with each of the 27 images paired with each direction twice).

### Behavioural analysis

For the analysis of the behavioral illusion quality rating and emotional arousal ratings during the fMRI task, separate 3 (MAE Direction: approach, recede, static) X 3 (Emotion: negative, neutral, positive) repeated-measures analyses of variance (ANOVAs) were conducted. Assumptions of sphericity were tested with a Mauchly’s test, with application of a Greenhouse-Geisser correction when necessary. Furthermore, to assist in interpretation of nonsignificant results, a subsequent Bayes Factor ANOVA with identical structure was conducted. Bonferroni corrections were performed on each set of follow-up comparisons to account for multiple comparisons. All behavioural analyses were conducted with the statistical software R (R Core Team, 2013) with the package BayesFactor.

### Imaging acquisition and analysis

#### MRI acquisition and preprocessing

Subjects were scanned during the task using a 3-Tesla Siemens Magnetom Prisma MRI scanner with a 32-channel head coil. fMRI images were taken with a T2*-weighted gradient-echo echo-planar imaging sequence (repetition time [TR] = 1,250 ms, echo time [TE] = 30 ms; field of view [FOV] = 192 mm, 96 x 96 matrix; multi-band acceleration factor = 3). All scanner images were acquired during a single scanning session. For all functional runs during the experimental task, complete brain coverage was obtained with 57 interleaved slices of 2-mm isovoxel resolution. A series of 268 functional images were collected during the V5/MT+ localizer run, and 456 for each experimental run. A whole-brain, high resolution T1-weighted anatomical scan was obtained between the functional localizer scan and the emotional MAE runs (TR = 2,300 ms, TE = 4.25 ms; FOV = 25.6 cm, 192 axial slices; voxel size = 1-mm isovoxel resolution; 256 x 240 matrix).

##### V5/MT+ localizer preprocessing

Analysis of the localizer scan was conducted with traditional univariate approaches. Regressors of interest were generated for both the dynamic and static visual conditions. These task-based regressors were modelled as block events with a duration of 18 s using AFNI’s default hemodynamic response function (i.e., 3dDeconvolve, [BLOCK 18,1]). In addition, the six parameters derived from motion correction (3 translations and three rotations) were included as regressors of no interest. The general linear model resulted in a β coefficient and t value for each voxel during both dynamic and static image presentation.

##### Experimental task preprocessing

Analysis of the fMRI data was conducted by using Analysis of Functional NeuroImages (AFNI v2016) software (Cox, 1996) at both the individual and group levels. To correct for motion, all volumes were registered to the functional volume acquired closest in time to the anatomical scan. The dataset for each participant was spatially smoothed (using an isotropic 4-mm, full-width, half-maximum Gaussian kernel). Time series data were normalized by dividing the signal intensity of a voxel at each time point by the mean signal intensity of that voxel for each run and multiplying the result by 100. Thus, resultant regression coefficients represent the percent signal change from the mean activity. For univariate analyses in the primary experimental task, nine regressors of interest were generated for the emotional MAE test phase, modeling the presentation time course for each of the nine conditions of interest (target image: 3 MAE directions X 3 emotional categories). To factor out the hemodynamic responses from the motion adaptation stimuli, three regressors of no interest were generated from the three kinds of adaptation periods (i.e., contracting, expanding and alternating motion concentric circles). One additional regressor was created to factor out the hemodynamic response related to the subjective rating questions (illusion quality and arousal). These task-based regressors were modelled by convolving box-car functions with AFNI’s default hemodynamic response function and the ANFI function 3dDeconvolve. In addition, to account for low-frequency temporal drift in the signal, additional regressors of no interest modeled a linear drift and a quadratic trend for each time series and to account for head motion, the six parameters derived from motion correction (3 translations and three rotations) also were included as regressors of no interest. Note, a separate control set of control analyses were performed on the motion data to examine participant motion across conditions. These included a 3 (direction) X 3 (emotion) rm-ANOVA conducted on each the six motion parameter parameters. No significant effects were identified (all *p* > 0.09). The general linear model resulted in a β coefficient and t value for each voxel and task regressor.

To facilitate multivariate analyses for similarity within conditions, two additional convolutions were performed, again with the AFNI function 3dDeconvolve. The first fit the hemodynamic response function to each motion-aftereffect regressor for all six experimental runs independently, while a single regressor modeled motion videos across run and direction thus resulting in 55 unique β coefficients (6 runs X 9 MAE conditions of interest + 1 motion video regressor). A second convolution was required to minimize the mathematically necessary anticorrelation of β coefficients resultant from fitting temporally adjacent and locked conditions. This fit the hemodynamic response function to each adaptation regressor for all six experimental runs independently, while a single regressor modeled MAE periods across run and direction thus resulting in 19 unique β coefficient (6 runs X 3 adaptation conditions of interest + 1 MAE regressor). All subsequent multivariate analyses were conducted on the run specific β coefficients. All six motion parameters described above regressors were included in both. To facilitate group analyses each individual’s data were transformed into the standard MNI space. For exploratory, whole brain analyses of the main experimental task, please see Supplemental Material S2 and Supplemental Table ST2.

#### Functional ROIs

##### Individually defined ROIs: V5MT+

To assess the impact of emotion on human visual motion processing, we targeted areas canonically implicated in these processes. Specifically, initial ROI analyses focused on functionally defined V5/MT+ regions identified by an independent localizer scan at the individual subject level. Given the relatively small volume of this region, and the expected inter-subject variability in brain morphology (Huang et al., 2019), defining this ROI independently for each participant allowed for maximal sensitivity in its interrogation. Swallow et al. (2003), a multistage process was used to identify the V5/MT+ complex at the single-subject level. First, general linear models were used to contrast single subject activity during the presentation of moving versus stationary stimuli in the functional localizer task. At a threshold of *p* < 0.001, a significant cluster containing V5/MT+ was contiguous with significant activation in additional early visual areas, including V1, V2, and V3 in 18 of 19 participants. To remove visual areas that are not specifically motion-sensitive regions (Tootell et al., 1995b), thresholds were adjusted until the resulting ROIs appeared the appropriate size (<1,000 mm^3^) and location of V5/MT+ as canonical descriptions of the area (Tootell et al., 1995b; Dumoulin et al., 2000). For full description of single subject V5/MT+ ROIs, see Supplemental Table ST3. Subsequently, these areas were used as functionally derived ROIs and applied to the MAE task to facilitate both univariate and multivariate analyses. For the univariate analysis, a 3 (MAE Direction; approaching, receding, static) X 3 (Emotion: negative, neutral, positive) repeated measures ANOVA was conducted on the percent signal change data derived from the left and right V5/MT+. Assumptions of sphericity were tested with a Mauchly’s test, with application of a Greenhouse-Geisser correction when necessary. Multivariate analyses are described below.

##### Group-derived ROIs: V5/MT+ localizer scan

To assess the impact of emotion on human visual motion processing in areas beyond V5/MT+ ROIs, a series of group-based, whole-brain analyses were conducted on the functional localizer data to identify general neural regions that were modulated by contrasting motion versus stationary stimuli. These allowed an exploratory investigation of motion-sensitive regions identified independent of our primary experimental task as the localizer was not optimized for this purpose (see Vanduffel et al., 2001 for alternatives). Data were thresholded to allow for the identification of group-derived bilateral V5/MT+ clusters noncontiguous with early visual areas (*p* < 0.0005; surviving correction to *p* < 0.01 through a spatial clustering operation performed by 3dClustSim –acf, v2016; Cox et al., 2017). All other surviving clusters were subsequently isolated as independent ROIs (Table 1). To investigate task-dependent activity within these ROIs, the percent signal change from each ROI was extracted for each condition of interest during the primary experimental task (i.e., during emotional MAE). To determine the extent to which activity within these ROIs were driven by both the presence of MAEs and emotional content of a stimulus, a 3 (MAE Direction: approaching, receding, static) X 3 (Emotion: negative, neutral, positive) repeated-measures ANOVA was conducted. Assumptions of sphericity were tested with a Mauchly’s test, with application of a Greenhouse-Geisser correction when necessary. Follow-up paired *t*-tests were conducted to investigate any significant effects.

**Table 1:** Group-derived ROIs identified in Motion vs. Still contrast

Contrast	R/L	Location	BA	X	Y	Z	Vol. (mm^3^)	Figure Reference
Motion > Still	R/L	Cuneus/MOG/LG	19	4	-90	5	16495^a^	4 (red)
	R	MTG/MOG	37	42	-62	6	441^b^	4a (orange)
	L	MTG/MOG	19/37	-43	-78	6	415^c^	4a (yellow)
Still > Motion	R/L	Precuneus/pCC/SPOC	31	9	-67	23	519	4b (green)
	L	PhG	39	-23	-50	5	516	4a/c (light blue)
	R/L	Cuneus/Precuneus	18/19	2	-77	32	286	4d (dark blue)
	R	PreCG	4	20	-26	62	156	4e (purple)

^a^ This region overlaps with the individually defined V5/MT+ ROIs (Fig. 3A) for two of nineteen subjects, for a total overlapping volume of 91mm^3^ (0.55%)

^b^ 376mm^3^ of this region (85.26%) overlaps with the individually defined V5/MT+ ROIs (Fig. 3A) of at least one subject

^c^ 195mm^3^ of this region (49.99%) overlaps with the individually defined V5/MT+ ROIs (Fig. 3A) of at least one subject

Significant clusters are thresholded at *p* < 0.0005 (corrected to *p* < 0.05)

MOG = middle occipital gyrus; LG = lingual gyrus; MTG = middle temporal gyrus; PhG = parahippocampal gyrus; pCC = posterior cingulate cortex; PreCG = precentral gyrus

XYZ are Talairach coordinates and refer to centre of mass

#### Structural ROIs

To assess the representation of motion and emotion in regions processing in visual motion-areas and to be able to compare this to patterns of representation in the canonical dorsal and ventral visual pathways, we generated four additional regions of interest to be used in multivariate analyses of our main experimental task. Three bilateral regions of interest (ROIs) were generated from the standard anatomical atlas (MNI_caez_ml_18) implemented in the Analysis of Functional NeuroImages (AFNI) software package (Cox, 1996) covering primary visual cortex (V1), ventral visual cortices (vVS), and dorsal visual cortices (dVS). While the extent of coverage of these ROIs is not ideal for investigation of specific subregions in these streams, it is meant to facilitate allowing investigation of multivariate representation of information processed within the broad divisions of visual processing pathways (see Supplemental Figure SF1 and Supplemental Table ST4 for a full anatomical descriptions of these ROIs). An additional ROI was derived from the individual subject V5/MT+ localizer scan. This ROI was defined by overlaying all individually defined V5/MT+ ROIs (see Supplemental Table ST2) to create a compound ROI of the area using the AFNI function 3dcalc. These regions (vVS, dVS, and V5/MT+) were chosen due to their role in visual classification (Kanwisher et al., 1997; Kravitz et al., 2013), visual guidance of behaviour (Milner and Goodale, 1993; Brogaard, 2012; Cloutman, 2013), and motion perception (Tootell et al., 1995b; Born and Bradley, 2005; Smith et al., 2006) respectively.

#### RSA and pattern component specification

To identify and compare the representational pattern elicited by the experimental conditions, representational similarity analysis (RSA; Fig. 1B) was performed by using the PyMVPA Python package (Hanke et al., 2009). Pattern component analyses followed procedures outlined in Kryklywy et al. (2023). For each participant, a vector was created containing the spatial patterns derived from β coefficients from each voxel related to each condition in each ROI. Pairwise Pearson coefficients were calculated between all vectors of a single ROI, resulting in a similarity matrix containing correlations across all 12 conditions (3 valence categories X 3 afterimage categories + 3 motion videos) for each participant. Fisher transformations were performed to ensure similarity metrics were normally distributed before comparisons between participants.

Representational patterns of interest (POIs) were generated to represent the similarity matrices that would be observed in the experimental data if it were to be a perfect representation of one of thirteen distinct potential sources of information related to the visual or emotional experience of the trial (see POI Glossary in Table 2 for details). Specifically, POIs were constructed for six visual constructs and seven valence-related constructs. For visual constructs, the POIs modelled included: 1) perceived approach, 2) perceived recession, 3) general perceived motion with a unique static representation), 4) general perceived motion without a unique static representation, 5) linear motion direction effects (approach-related activity as anticorrelated with receding), and 6) image identity (i.e., the specific group of images used). Valence POIs related to the emotional quality of the images viewed. These were defined independently to reflect both the valence of the image, as well as the valence of the trial outcome (e.g., a receding negative image is a positive outcome). The valence-based POIs modeled: 7) all valenced images (i.e., represents positive or negative stimuli collectively as distinct from neutral stimuli), 8) positive images (versus negative or neutral collectively), 9) negative images (versus positive or neutral images collectively), 10) a linear image valence spectrum (i.e., positive images anticorrelated with negative images), 11) positive outcome, 12) negative outcomes, and 13) a linear outcome valence spectrum (adaptive outcomes [positive stimulus approach or negative stimulus receding] anticorrelated with maladaptive outcomes [positive stimulus receding or negative stimulus approaching]).

**Table 2:**
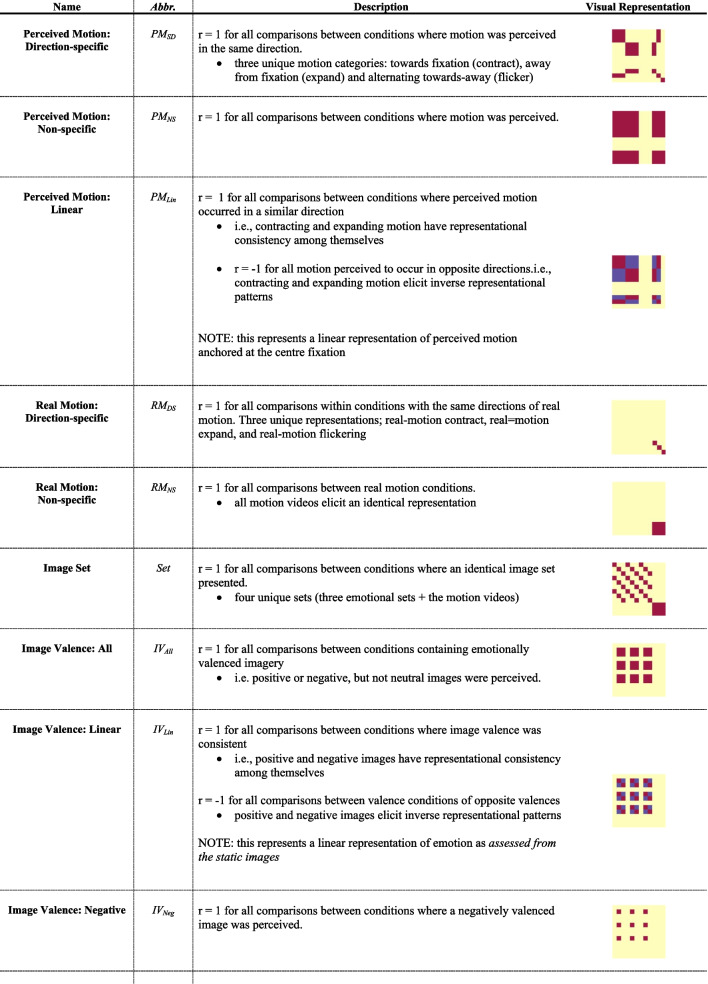

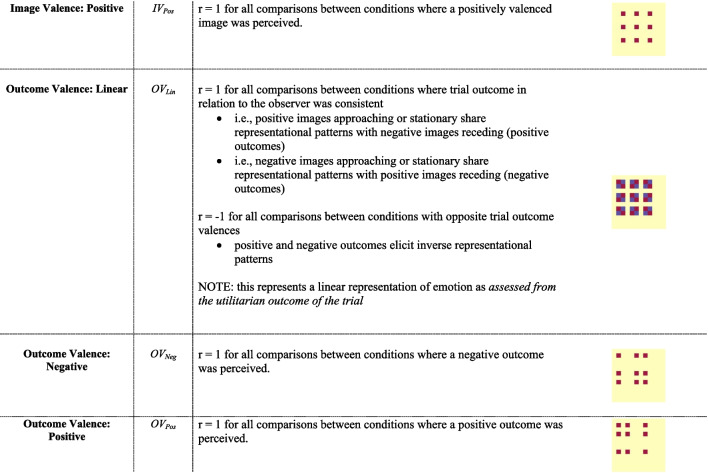
POI Glossary

^*^General matrix structure can be found in Fig. 1B; red represents *r* = 1, blue represents *r* = -1 and yellow represents *r* = 0

Pattern component modelling, a novel neuroimaging technique (Diedrichsen et al., 2018; Kryklywy et al., 2021a; 2023), was conducted to identify and fit the POIs that best described observed similarity in each of the four ROIs. To avoid overfitting of the data by the PCM procedures, we performed a Monte-Carlo cross validation (Picard and Cook, 1984) (MCCV) with 1,000 iterations. For each iteration, initial analyses were conducted on a randomly selected (RS) subset of ten participants (RS = 10), with results validated against the remaining nine participants (the “hold-out”; HO = 9). These parameters were chosen to maximize cross-validation performance by minimizing CV-variance while maximizing model selection accuracy given our initial sample size (Arlot and Celisse, 2010; Valente et al., 2021).

In the current experiment, many of the modeled representational patterns of interest (POIs) contained overlapping similarity; thus, a traditional regression including all potential components as predictors would be insufficient to identify those that are most informative. To accommodate this, PCM conducted in data from each RS implemented iterative Bayesian Information Criterion (BIC) with an uninformed *greedy best-first search* (*GBFS*) algorithm (Doran and Michie, 1966) to determine the POI combinations that best explained the observed correlation. First-level model fitting identified the single POI most predictive of the observed similarity patterns. This was then tested in combination with all remaining POIs to which combination led to the greatest improvement in model fit, and this process was repeated until the addition of no other POIs led to improved fit. Improved fit due defined as a ΔBIC > 2 (Fabozzi, 2014). If multiple POI combinations at a given search level resulted in a statistically equivalent “best fit” (i.e., ΔBIC from the absolute best fit < 2), all equivalent paths were extended to completion (Fabozzi, 2014). POIs present in the completed path with the lowest end BIC score were identified as contributing to representations in that region. Correlation matrix transformations were performed by using Matlab r2019a (The MathWorks, Natick, MA), and BIC analyses were conducted with the lme4 statistical package in R 4.0.2 (R Core Team, 2013). Custom code for the PCM analyses is available in the R package PCMforR (Kryklywy et al., 2021b). This approach allowed for the identification of multiple sources of information as they simultaneously contribute to observed representational patterns, rather than any single information construct. By iteratively performing these analyses (1,000 samples of n = 10), we were able to identify the proportion of subsamples identifying a specific POI as contributing to the observed similarity patterns in the experimental data (POI identification rate). POI identification rates were compared to chance identification (average # of POIs identified per iteration / total # of POIs for each ROI.

## Results

### Behavioural results

**Fig. 2:**
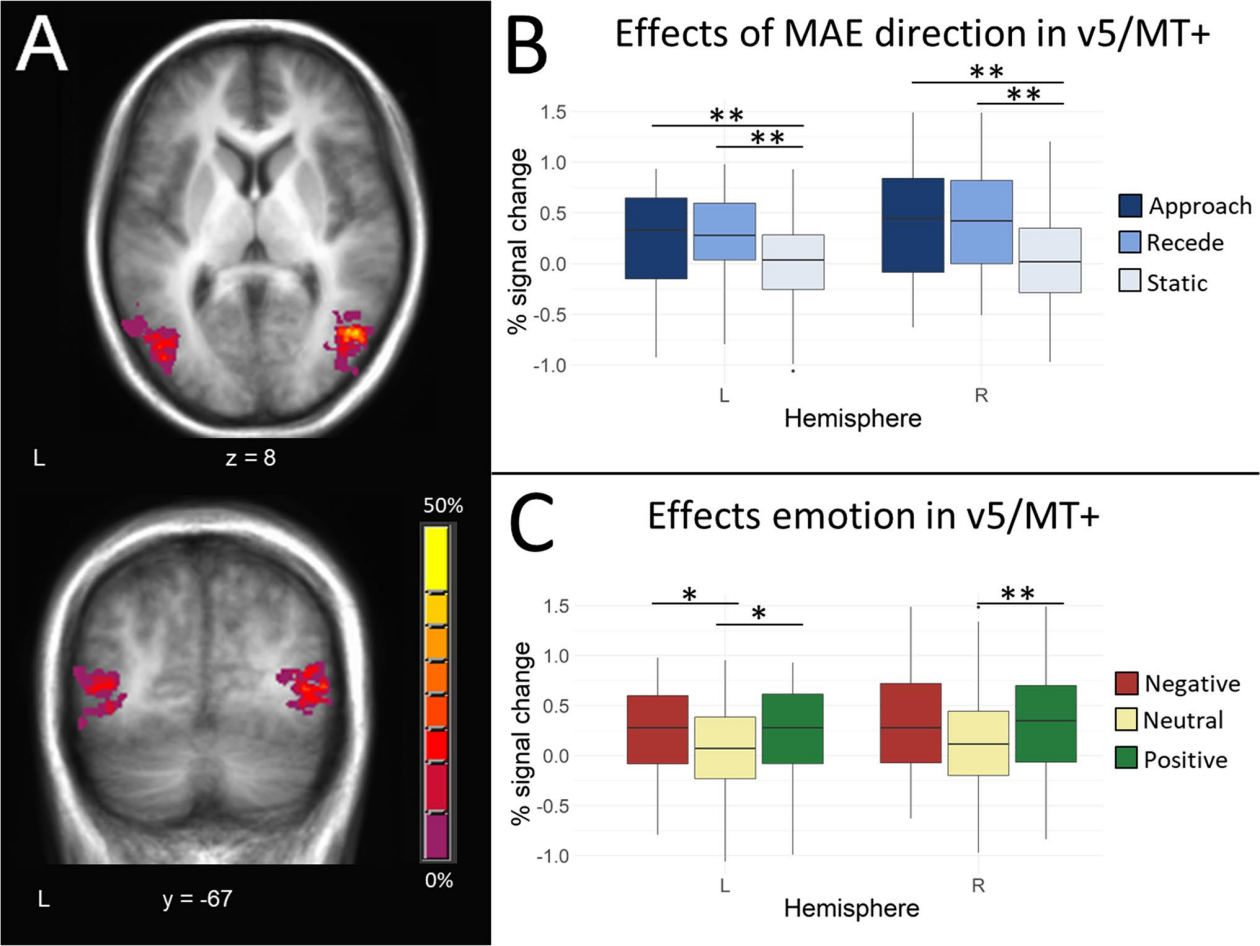
Behavioural effects of MAE direction and emotion. (**A**) The direction of illusory motion was found to significantly impact illusion quality ratings. Both approaching and receding MAEs elicited significantly higher quality of illusion than static afterimages. (**B**) Ratings of perceived arousal were modulated by emotion and showed a significant motion X direction interaction. Main effect of emotion is indicated on the figure legend. For all plots, bottom/top of boxes indicate 25^th^ and 75^th^ percentile respectively; whiskers extend smallest/largest value (no further than 1.5 X interquartile range). *p < 0.05; **p < 0.01; ***p < 0.001

A 3 (MAE Direction) X 3 (Emotion) repeated-measures ANOVA, paired with a Bayes factor ANOVA of similar structure were conducted on the illusion quality ratings (Fig. 2A). These yielded a significant main effect of MAE direction (F_(1.48,26.71)_ = 52.06 *p <* 0.001) with decisive evidence for the alternate hypothesis (H_1_; BF_10_ > 100). Critically, the approaching and receding after-effects were rated to be significantly more robust (i.e., created a stronger motion illusion) than the static aftereffects (t_(18)_ = 8.50; *p <* 0.001 and t_(18)_ = 4.78; *p <* 0.001 respectively; Fig. 2A). In addition, approaching images were seen as having significantly more apparent motion than receding images (t_(18)_ = 6.26; *p <* 0.001). No significant main effect of emotion (F_(1.12, 20.20)_ = 0.60, *p* > 0.10) emerged, consistent with strong evidence for the null hypothesis (BF_10_ = 0.070 ± 2.35%). In addition, the ANOVA identified no MAE direction X emotion interaction (F_(4, 72)_ = 2.22, *p* = 0.075), supported by strong evidence for the null hypothesis (BF_10_ = 0.07 ± 3.01%).

A 3 X 3 repeated-measures ANOVA and Bayes factor ANOVA of similar design were applied to the emotional arousal data (Fig. 2B). These yielded a significant main effect of emotion (F_(2,36)_ = 54.29, *p <* 0.001), with decisive evidence for the H_1_ (BF_10_ >100). This effect was characterized by significantly greater emotional arousal ratings for both negative and positive images compared with neutral images (t_(18)_ = 7.50; *p <* 0.001 and t_(18)_ = 4.53; *p <* 0.001 respectively) as well as significantly higher emotional arousal ratings for negative images compared with positive images (t_(18)_ = 3.27; *p* = 0.007). No main effect of MAE direction was identified in the arousal data (F_(2,36)_ = 1.724, *p* = 0.19), with strong evidence to support H_0_ (BF_10_ = 0.064 ± 1.11%). A significant MAE direction X emotion interaction emerged (F_(4,72)_ = 4.78, *p* = 0.002), albeit unsupported by Bayesian analyses (BF_10_ = 0.092 ± 3.06%). A subsequent series of paired *t*-tests (all reported *p* values subject to Bonferroni correction) determined the interaction effect was driven by increased arousal during negative and positive image trials compared to neutral image trials in all motion directions (all *p*s <0.001), and significantly greater arousal for negative image trials compared to positive image trials during approaching (*p* = 0.016), *but not* receding or static trials (*p* = 0.12 and *p* = 0.25 respectively; Fig. 2B).

### Univariate imaging results

#### Individually Defined ROIs: V5/MT+

**Fig. 3:**
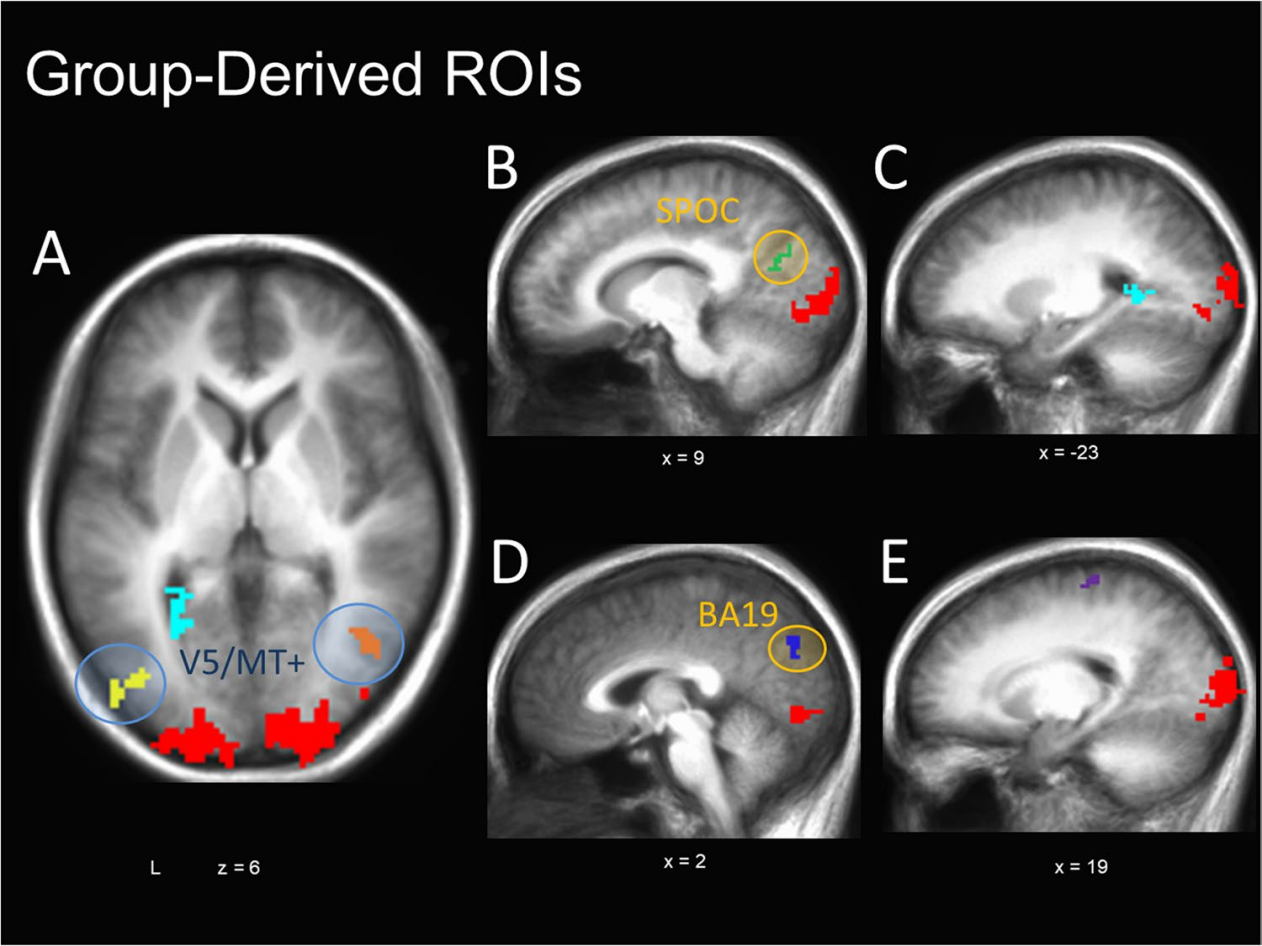
V5/MT+ responses to emotion and perceived MAE direction within individually defined ROIs. (**A**) Spatial overlap of subject ROIs for bilateral V5/MT+ (Participant #3). (**B**) Activity in bilateral V5/MT+ across participants was modulated as a function of illusory motion, with both approach and recede MAE trials eliciting significantly more activity than static control trials. (**C**) Activity in this region was also modulated as a function of emotion. Both positive and negative trials elicited significantly more activity than neutral trials. No direction by emotion interaction was observed. Error bars represent standard error of the mean. For all plots, bottom/top of boxes indicate 25^th^ and 75^th^ percentile respectively; whiskers extend smallest/largest value (no further than 1.5 X interquartile range). *p < 0.05; **p < 0.01; ***p < 0.001

Initial analyses addressed whether emotional images augmented activity in V5/MT+, similar to their effects on areas traditionally associated with the ventral visual stream. Individually defined ROIs generated from the V5/MT+ localizer scan (Fig. 3A; for group overlap in V5/MT+ identification) were applied to the data from the emotional MAE task (an independent data set), and a 3 (MAE Direction) X 3 (Emotion) repeated measures ANOVA was conducted on the percent signal change data derived from the left and right V5/MT+.

A main effect of MAE direction (right: F_(1.48,26.59)_ = 13.28, *p <* 0.001; left: F_(2,36)_ = 3.85, *p* = 0.031; Fig. 3B) emerged, with decisive evidence against the null hypotheses observed in the right hemisphere (BF_10_ > 100) and inconclusive evidence for or against the null hypothesis observed in the left hemisphere (BF_10_ = 0.48 ± 0.89%). As expected, there was enhanced V5/MT+ activation to approaching (right: *p* = 0.0033; left *p* = 0.0073) and receding trials (right: *p* = 0.0028; left *p* = 0.0056) compared with static trials, and no difference in activation between approaching and receding stimuli (right: *p* = 1; left *p* = 1). More importantly, a significant main effect of emotion emerged in both hemispheres (right: F_(1.44,25.99)_ = 6.42, *p* = 0.009; left: F_(2,36)_ = 3.81, *p* = 0.032; Fig. 3C), again supported by substantial evidence against the null hypotheses (right BF_10_ = 4.32 ± 1.18%; left BF_10_ = 5.47 ± 0.73%). This was characterized by increased activity for trials containing a positive target image compared with trials containing a neutral target image (right: *p* = 0.0095 0.001; left *p* = 0.012) and increased activity in left V5/MT+ to trials with a negative compared with a neutral image (*p* = 0.023) but not in right V5/MT+ (*p* = 0.12). No significant differences were noted between the emotional categories (negative vs. positive) in either hemisphere (both *ps* = 1). No significant MAE direction X emotion interaction was found (right: F_(4,72)_ = 0.58, *p =* 0.832; left: F_(4,72)_ = 0.58, *p* = 0.68) with strong support for the null hypothesis (right BF_10_ = 0.055 ± 4.67%; left BF_10_ = 0.080 ± 5.11%). Note p values for all paired comparisons have been subject to Bonferroni correction. By overlaying individually defined V5/MT+ ROIs, a compound V5/MT+ ROI was obtained that extended over motion-sensitive MT regions for all participants (see Section "Structural ROIs"). This mask was created for use in subsequent multivariate statistics.

#### Group-derived ROIs: V5/MT+ localizer scan

**Fig. 4:**
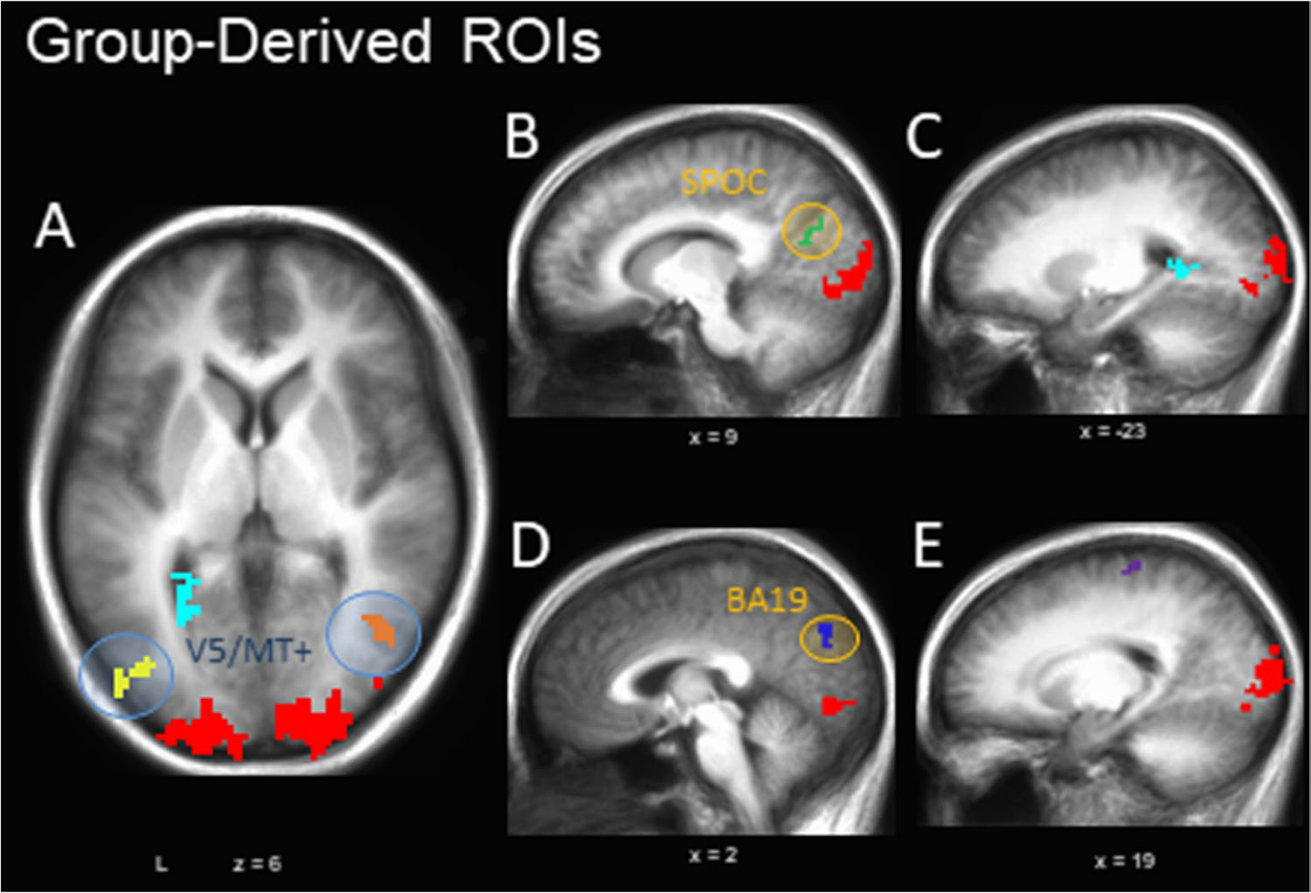
Group-defined motion-selective ROIs. Group-defined ROIs were identified by contrasting neural activity in motion versus static viewing conditions during the functional localizer, identifying regions of the occipital and middle temporal lobes (ventral visual stream) which respond preferentially to moving, but not static imagery (green). Independent bilateral V5/MT+ regions were identified separate from a large ventral visual cluster (blue circles). In addition, areas of the dorsal occipital lobe (SPOC and dorsal BA18/19; orange circles), parahippocampal gyrus, and post-central gyrus displayed preferential activation to the static control than the motion condition (yellow)

A paired *t*-test was performed on whole-brain localizer scan data to identify widespread regions across participants with differential activation for moving versus static pictures, including potential regions associated with the dorsal visual stream (Table 1; Fig. 4). This isolated bilateral clusters of activation located along V5/MT+ (Fig. 4A), as well as widespread early visual processing areas. In additional, two separate regions of dorsal occipital lobe were identified, including portions of the superior parieto-occipital cortex (SPOC; Fig. 4B), which likely corresponds to the motion-selective visual area V6 (Pitzalis et al., 2015), and the cuneus (BA19 /prostrita; Mikellidou et al., 2017, Fig. 4D), as well as one region along the parahippocampal gyrus (Fig. 4C), and one on the precentral gyrus (possibly a motor-confound; Fig. 4C). These ROIs were applied to the experimental task data, and a 3 (MAE Direction) X 3 (Emotion) repeated-measures ANOVA was conducted on the percent signal change data extracted from each. Therefore, this analysis examined the impact of illusory motion and emotion on signal in neural regions identified as being responsive to actual motion in the original localizer.

Consistent with the individually defined V5/MT+ ROIs, both left and right group-defined V5/MT+ ROIs were modulated significantly by the emotional quality of the image (right: F_(2,36)_ = 10.75, *p <* 0.001, BF_10_ = 53.0 ± 0.96%; left: F_(2,36)_ = 26.07, *p <* 0.001, BF_10_ > 100). This effect was characterized by significantly greater activity for positive and negative stimuli compared with neutral stimuli (right V5/MT+: *p* < 0.001 and *p* = 0.009 respectively; left V5/MT+: both *p* < 0.001) and no difference between positive and negative stimuli (*p* = 0.14). A significant main effect of MAE direction was identified within right V5/MT+ (F_(2,36)_ = 13.43, *p <* 0.001, BF_10_ > 100), characterized by greater activity for approaching and receding stimuli compared with perceptually stationary stimuli (*p <* 0.001 for both contrasts) and no difference between approaching and receding stimuli (*p* = 0.22). No significant main effect of MAE direction was identified in left V5/MT+ (F_(2,36)_ = 1.49, *p* = 0.24), consistent with substantial evidence in favour of the null hypothesis (BF_10_ = 0.20 ± 1.23%)). No significant MAE direction by emotion interaction observed in either hemisphere (right: F_(4,72)_ = 0.674, *p* = 0.61; left: F_(2.68,48.3)_ = 0.78, *p* = 0.83), supported by strong evidence for the null hypothesis (BF_10_ = 0.068 ± 1.53%).

Investigation of a large occipital cluster identified as motion sensitive in the V5/MT+ Localizer scan (including bilateral regions of both early and ventral visual cortices with the cluster extending into V1, V2, LOC; Table 1; Fig. 4) found it to display similar responses to emotional content as observed in V5/MT+ (ME Emotion: F_(1.401,25.215)_ = 6.40, *p* = 0.0042, BF_10_ = 55.59 ± 0.64%). By contrast, main effects of emotion were not observed in other visual regions identified in the functional localizer as motion-sensitive, including dorsal visual regions and visual regions less defined within the dorsal-ventral framework (SPOC and BA 18/19 respectively; Fig. 4; all *p*s > 0.20, BF_10_ < 0.23). Furthermore, no significant main effect of MAE direction or MAE direction by emotion interaction was identified in either of these visual regions (all *ps >* 0.25, BF_10_ < 0.28). No significant main effects or interaction involving emotion were identified within the precuneus (all *p*s* >* 0.09, BF_10_ < 0.40) or parahippocampal gyrus (all *p*s* >* 0.27, BF_10_ < 0.3).

### Multivariate analyses

**Fig. 5:**
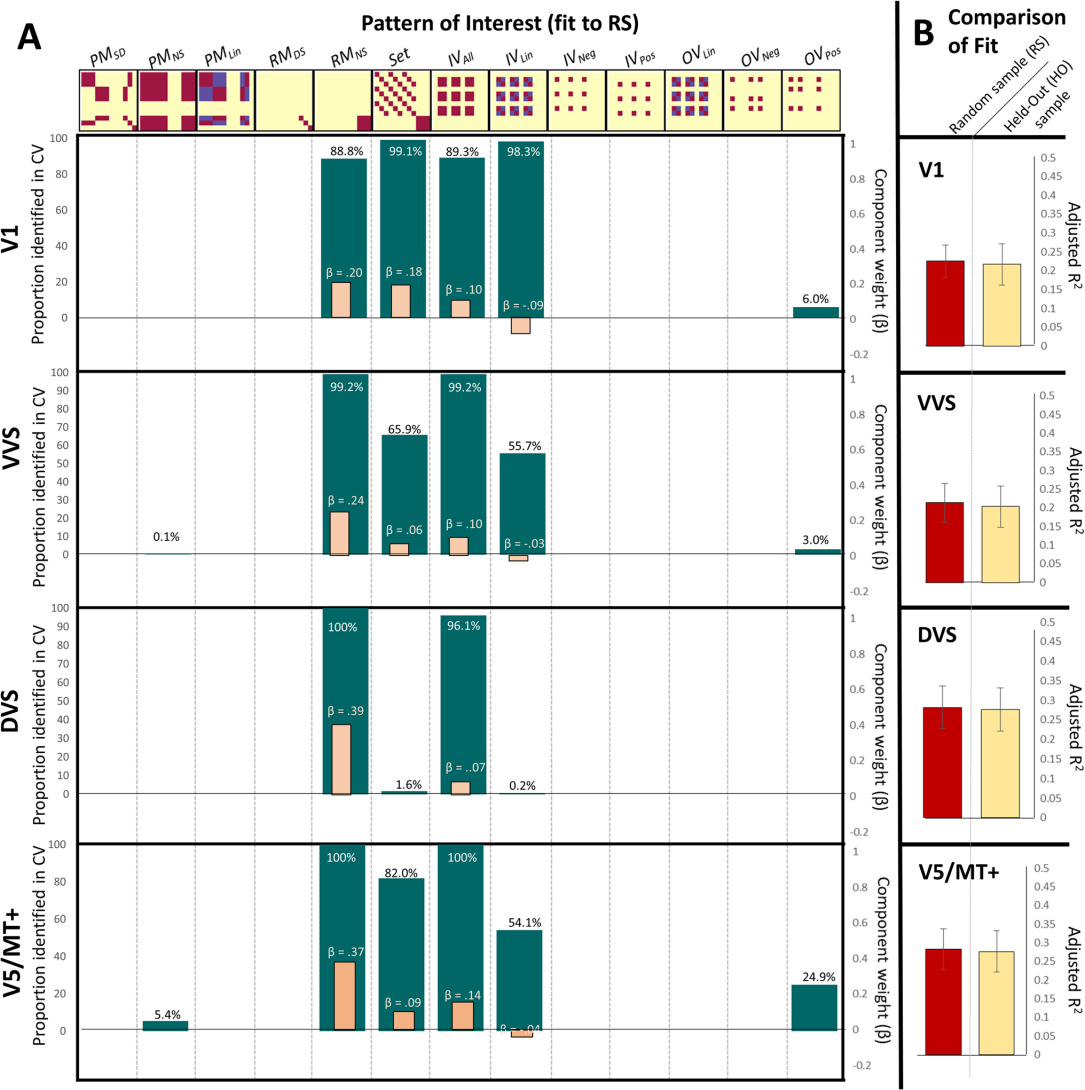
Monte-Carlo CV of PCM analyses. Pattern component modelling was performed through 1,000 iterations Monte-Carlo cross-validation procedure with a random sample (RS) of 10 participants and nine held-out (HO) participants. (**A**) Potential representational pattern of interest (POIs), reflecting operationalized states of experience (Table 2), are presented along the top of the figure, with results from each ROI present below. The proportion of MCCV iterations identifying each pattern as a contributing to observed representational patterns in each brain region is indicated by the teal bars. The average β value for components for only those POIs contributing to the representational pattern at a rate significantly greater than chance is presented overlaid on the relevant component (peach bar; scale to the right). (**B**) Average POI fit in the RS and HO. Red bars (right) indicate the proportion of variability in the RS attributed to the identified POI identified in the PCM analyses. Light yellow bars (left) indicate the proportion of variability in the HO attributed to the identified POI. POI components, and their relevant weight were identified in the RS only. No significant differences emerged in R^2^ between the RS and HO for any of the tested brain regions. Error bars represent standard deviation for each MCCV distribution. Abbreviations for POIs identified at a rate significantly greater than chance: Real motion, nonspecific (RM_NS_); Image set identity (Set); Image valence, all salience (IV_All_); Image valence, linear (IV_Lin_). Abbreviations for all noncontributing POIs can be found in Table 2

To identify the emotional properties of information represented across visual areas, we conducted Pattern Component Modelling, with a cross validation procedure (CV; for full details, see Table 3; Fig. 5). Results presented include 1) the strength of representation for *Patterns of Interest* (POIs) identified as contributing to similarity representations in the random sample, 2) the subsequent fit of these POIs when applied as predictors to similarity patterns observed in the help out participants. This technique allows for the identification of predicted POIs in neural regions subserving multiple, or integrative functions, as well as from representational states prone to signal noise. Specifically, it allows the interrogation of representational patterns that may not be the dominant representations in the area (i.e., they explain low overall variance) yet do make significant contribution to the overall representational patterns.

**Table 3:** Cross validation–Average values average βs are presented only for POIs identified at a level significantly greater than chance (i.e., proportion of simulations POI is identified > (Average # of contributing POIs / Total POIs tested))

	Av. n-path	Pattern of Interest identification –% of Simulations (mean contributing β*; *n* = 19)													HO Fit (*n* = 9, df = 700)		
ROI		PM_SD_	PM_NS_	PM_Lin_	RM_DS_	RM_NS_	Set	IV_All_	IV_Lin_	IV_Neg_	IV_Pos_	OV_Lin_	OV_Neg_	OV_Pos_	R^2^	*P value*	Recon. β
**V1**	1.145	0 -	0 -	0 -	0 -	88.8 (0.196)	99.1 (0.181)	89.3 (0.096)	98.3 (-0.090)	0 -	0 -	0 -	0 -	0.6 -	.239	1.42e-29	1.004
**VVS**	1.190	0 -	0.1 -	0 -	0 -	99.2 (0.236)	65.9 (0.064)	99.2 (0.097)	55.7 (-0.030)	0 -	0 -	0 -	0 -	0.3 -	.226	7.87e-23	1.001
**DVS**	1.020	0 -	0 -	0 -	0 -	100 (0.392)	1.6 -	96.1 (0.073)	0.2 -	0 -	0 -	0 -	0 -	0 -	.294	5.31e-31	0.990
**MT**	1.367	0 -	5.4 -	0 -	0 -	100 (0.370)	82 (0.099)	100 (0.150)	54.1 (-0.038)	0 -	0 -	0 -	0 -	24.9 -	.300	1.06e-34	0.979

#### RSA with pattern component modeling

*V1:* Representations in V1 were explained by a combination of POIs modelling the general presence of actual visual motion of the stimuli (*β*_*RMNS*_ = 0.196), the specific set of images observed (*β*_*Set*_ = 0.181), the emotional salience of the images (*β*_*IVAll*_ = 0.096) and the valence of the images represented along a linear spectrum (*β*_*IVLin*_ = −0.090). Together, these weighted components predicted an average of 23.9% of the variance in the remaining held-out participants. (*R*^*2*^ = 0.239, *F*_(1, 700)_ = 223.95, *p* < 0.001, β = 1.00). These results suggest that V1 represents multiple features of visual experience, including motion, valence, identity, and emotional salience.

##### Ventral visual structures

In a pattern similar to V1, the representations in the ventral visual stream (vVS) were significantly explained by POIs modelling varied visual features. Specifically, identified components included the general presence of actual visual motion in the stimuli (*β*_*RMNS*_ = 0.236), the specific set of images observed (*β*_*Set*_ = 0.064), the emotional salience of the images (*β*_*IVAll*_ = 0.097) and the valence of the images along a linear spectrum (*β*_*IVLin*_ = −0.030). A reconstructed component generated from these weighted components predicted an average of 22.6% of the variance in the remaining held-out participants (*R*^*2*^ = 0.226, *F*_(1, 700)_ = 209.43, *p* < 0.001, β = 1.00). The similarity of the POIs that best explain the representations in V1 and vVS, with their similarity of representational strength (i.e., beta weights) suggest a continuity of processing for a number of visual features between these regions.

##### Dorsal visual stream

In the dorsal visual stream (dVS), POIs modelling the general presence of actual visual motion in the stimuli (*β*_*RMNS*_ = 0.392) and the emotional salience of the images—as defined by standardized ratings (Lang et al., 2008)—were found to be represented (*β*_*IVAll*_ = 0.073). A recombination of these weighted components explained and average of 29.4% of variance in the held-out participants (*R*^*2*^ = 0.294, *F*_(1, 700)_ = 302.28, *p* < 0.001, β = 0.99). These results suggest that dVS is important in representing information about motion in any direction as well as image salience, but, importantly, does not represent specific features for static visual identification (i.e., no representation for image identity), or any specific valence-based information (positive vs. negative) of the trials.

##### V5/MT+

With a component combination similar to V1 and vVS, and divergent from dVS, representations in V5/MT+ were significantly explained by a combination of POIs modelling varied visual features. Specifically, identified components included the general presence of actual visual motion in the stimuli (*β*_*RMNS*_ = 0.370), the specific set of images observed (*β*_*Set*_ = 0.099), the emotional salience of the images (*β*_*IVAll*_ = 0.150) and the valence of the images along a linear spectrum (*β*_*IVLin*_ = −0.038). Together, these weighted POIs predicted an average of 30% of variance in the held-out participants (R^2^ = 0.30, *F*_(1, 700)_ = 302.13, *p* < 0.001, β = 0.98). Of note, while the component patterns identified in this region were highly consistent to those in V1 and vVS, the representational weight of actual visual motion (*β*_*RMNS*_*)* was more consistent to that observed in dVS. This demonstrates that V5/MT+ is unique in that it represents both real visual motion and the valence of the images (dissociating between positive from negative valenced images). Additionally, the identification of valence-based processing by multivariate but not univariate approached suggest that these representations are coded by activity pattern changes across multiple voxels (Kriegeskorte et al., 2008), likely indicating population coding of this information (Chikazoe et al., 2014).

## Discussion

The present study investigated the impact of emotion on the motion processing region V5/MT+. Consistent with predictions, increased activity in this region was observed during motion aftereffects compared to static control conditions and for emotional compared to neutral images; however, in contrast to what was expected, no emotion-direction interactions were observed. Furthermore, both univariate and multivariate analyses demonstrated that the emotional valence of a visual stimulus changed the neural activity and observable representational patterns throughout the ventral visual stream, but not in dorsal stream areas responsive to visual motion (i.e., SPOC or dorsal BA18/19). Similarly, representational similarity analyses (RSA) conducted with theory-driven Pattern Component Modelling (PCM) determined that the representational profile of the V5/MT+ complex more closely aligned with those observed in primary and ventral rather than dorsal visual regions. Consistent with the univariate results, area V5/MT+ represented both motion and emotion, with these factors represented as independent, rather than integrated, components. Specifically, V5/MT+ houses valence-based representations of emotional content and image identity, consistent with those observed in early and ventral visual regions. Representation patterns in dorsal visual structures did not include valence or identity features but was instead limited to motion and general saliency. The strength of representation for real visual motion (i.e., the adaptation videos; *β*_*RMNS*_) in V5/MT+, however, was consistent with that observed in traditionally dorsal regions (and notably greater than in ventral structures), suggesting a similar degree of processing for visual motion across these regions. These results suggest that V5/MT+ is unique in its emotional visual processing. Its shares similar sensitivity to visual motion as the canonical dorsal stream yet responds to emotional information in a manner more consistent with ventral-visual processing. These results are interpreted with reference to the impact of emotion in the preparation for, and representation of, motion in visual processing areas.

### Motion modulation of emotional arousal

In the current study, emotional arousal ratings were modulated by both stimulus valence and direction of perceived motion relative to the viewer’s position. Approaching negative images were found to elicit the highest emotional arousal rating. This effect is in line with conceptualizations of arousal as a state designed to elicit biological preparedness, particularly the idea that proximal approaching threats place greater demands on behaviour than retreating ones (Panksepp, 1990; 1998; Mobbs et al., 2009; 2020; Abe et al., 2018) and that arousal increases with threat proximity (e.g., a tarantula; Mobbs et al., 2010). Behavioural analyses suggest that motion-specific effects of on arousal we limited to negative stimuli—consistent with previous work (Sato and Yoshikawa, 2007), increased arousal of neutral stimuli did not depend on the direction of motion. It is important to note that the principal manipulation in the current study relied on illusory motion, thus keeping other visual properties (e.g., size) constant, yet it was capable of impacting not only motion perception, but also emotional arousal ratings and activation in emotion-sensitive brain areas. This highlights the potential value of motion after-effects for examining how motion and changes in apparent stimulus proximity may interact with other stimulus features, such as emotional significance, to influence brain and behaviour.

### Neural responses to emotion

Large areas of the ventral visual stream dedicated to object perception (including inferior occipital and middle temporal lobe; Milner and Goodale, 2006) and primary visual cortices displayed valence-specific fluctuations in neural activity, and multivariate patterns associated with emotional content, which are both consistent with previous work (Lang et al., 1998; Morris et al., 1998; Vuilleumier and Driver, 2007). Of note, however, while efforts were made to statistically match many visual characteristics across valence categories (see Section "Stimuli and Apparatus"), the preference towards naturalistic images in the current work meant that exact matching was not possible. Thus, one should take appropriate caution when interpreting cross-valence results with additional work using scrambled images with control spectral components would be useful to further tease apart these effects. The observed effects, however, are consistent with initial predictions. Specifically, similar robust valence-based effects were observed across, bilateral V5/MT+, ventral visual areas an primary visual cortex, while substantially less emotional responsivity was observed in dorsal visual regions, despite the fact that this V5/MT+ is traditionally associated with dorsal stream processing (Buchel and Friston, 1997; Born and Bradley, 2005). Representational patterns in area V5/MT+ were found to be most strongly predicted by the general emotional salience of the images (POI: IV_All_), with highly correlated activational states for emotional images regardless of valence polarity. This suggests that this region may be particularly tuned towards integrating the emotional salience (Vuilleumier, 2005; Vuilleumier and Driver, 2007; Pessoa and Adolphs, 2010; Mitchell and Greening, 2012) of stimuli to its observer-centered movements.

Interestingly, the observation of enhanced processing V5/MT+ is not limited to objects of emotional salience but rather occurs with increased attentional salience of many forms (Huk et al., 2001). Thus, some of the observed effects may not reflect an emotion-specific changes to V5/MT+ processed but rather demonstrate how emotional information can leverage mechanisms of general attentional prioritization. Additional work may be needed to determine the emotion specificity of effect observed in this area. Either interpretation, however, is consistent with recent evidence that motion cues can facilitate object perception (e.g., aggressive movements facilitating the detection of threat in crowded visual scenes; van Boxtel and Lu, 2012; Parasuraman and Galster, 2013). Although we predicted little influence of emotion on dorsal visual processing (Kryklywy et al., 2013; Kryklywy and Mitchell, 2014; further supported from the current univariate analyses; Kryklywy et al., 2018), representational patterns reflecting the non-valenced emotional salience (POI: IV_All_) were observed in these regions. This incongruence between the current univariate and multivariate results may reflect either fundamentally different types of neural encoding detectable by these analyses (i.e., population coding vs. activation coding; see Kriegeskorte and Kievit, 2013; Kryklywy et al., 2018) and highlight the benefit of multipronged analytic approaches.

Limited emotional influence on univariate activation of dorsal visual regions was observed in the current work. While this is superficially incongruent with previous studies that have observed robust emotion-related effects to dynamic body and facial expressions in canonically dorsal areas (Lang et al., 1998; de Gelder et al., 2004; de Gelder and Hadjikhani, 2006; Grecucci et al., 2013; Goldberg et al., 2012; 2014; Engelen et al., 2015), it is important to note that previous work often provided clear conspecific social contexts in their emotional stimuli. By contrast, the current paradigm incorporated emotional visual scenes that consisted of objects *and* contexts. Some of these scenes involved humans interacting (e.g., laughing children), and others did not (e.g., snarling dog). While seemingly minor, it become important when considering the potential role of the dorsal stream in social response selection (Kong et al., 2021), and evidence from the current work demonstrating that emotional salience of images processed in earlier visual regions may still maintain representation in dorsal structures. Alternatively, a recent proposal of a third visual stream—one located along the middle and superior temporal gyrus and implicated in processing visual cues for social perception (Pitcher and Ungerleider, 2021)—may help to illuminate how our visual system may process social cues. Within this novel framework, the emotionality of *social information* may more efficiently communicate to dorsal processing regions, whereas the emotionality of *nonsocial cues* (as presented in the current work) stays relatively confined to ventral areas during visual processing. Additional work delineating the interconnectivity of this newly proposed pathway with canonical processing streams is required, however, before further interpretation of these results.

### Neural responses to motion

As predicted from previous work (Tootell et al., 1995a; Culham et al., 1999; Antal et al., 2004; Fawcett et al., 2007; Hogendoorn and Verstraten, 2013), the V5/MT+ complex displayed preferential response to apparent motion in an image. In addition, the traditional role of V5/MT+ in motion-based processing (Buchel and Friston, 1997; Born and Bradley, 2005) was supported by representational similarity analyses. Representation patterns observed in V5/MT+ were found to be driven in part by the *real* visual motion (i.e., distinct representational patterns for the adaptation videos) presented during the individual trials. Pattern component modeling analyses in this region, however, did not identify pattern components representing the *perceived* motion (i.e., no shared representational patterns between the adaptation videos and motion after-effects).

One possible explanation is that illusory motion is processed by a separate neural mechanism than real motion. Motion direction is encoded in the brain by cumulative activity across a population of neurons rather than by individual neural firings, (i.e., a population code; Georgopoulos et al., 1986; 1988; Maynard et al., 1999). Different patterns of visual motion may be represented by partially, or even nonoverlapping groups of neurons, rather than different levels of activity within a single population. Thus, the experience of a motion after-effect following sustained motion in one direction may be the result of *down-regulation*, or fatigue, of cell populations representing the initial direction of presented motion rather than an *up-regulation* of the cell populations representing the direction of the subsequent illusionary motion (Anstis et al., 1998). In this manner, the activation patterns representing real visual motion may not overlap with those representing congruent motion aftereffects and thereby would go undetected by similarity-based analyses. This mechanism of fatigue, however, has been challenged by evidence that motion-aftereffect storage persists beyond the expected time course for neural fatigue (Watamaniuk and Heinen, 2007) and that it can be generated from still photos with suggested motion (Winawer et al., 2008). Furthermore, this mechanism is challenged by the univariate analyses conducted in the current work. In these, activation in V5/MT+ was found to have *increased* activity during MAE conditions versus static image conditions. This suggests that a down-regulation of neural activity is unlikely and also indicates that whereas overall activation may change for individual voxels across motion aftereffects, the relative pattern of activity between voxels remains stable. At this point, it should be noted that delineating population-based encoding from activity V5/MT+ (or any other brain region) remains a notably challenging endeavour (Bartels et al., 2008). Activity in this region has been found to both increase and decrease is response to coherent motion (Becker et al., 2008), among other forms of heterogenic responding (Laycock et al., 2007; Kayser et al., 2010; Emmerling et al., 2016; Liu and Pack, 2017; Gaglianese et al., 2023). Thus, appropriate caution should be taken for all interpretation of the V5/MT+ results.

An alternative explanation to the neural fatigue theory to explain a limited representation of illusory motion in V5/MT+ can be evoked when considering the time course of modelled events in the current work. If real and illusory visual motion are coded by overlapping populations of neurons, it may be that the time course modeled in the representational similarity analyses extend beyond the extinction of the visual illusion, thus including stationary imagery as well. This may have resulted in diluted representational patterns of motion during aftereffect conditions and led to the reduced similarity observed between the illusory and real motion conditions. Canonical evidence of motion aftereffect phenomena suggests that this speed of decay is unlikely given the paradigm used (Hershenson, 1989). It may be instead that illusory motion, including the motion after-effect, is not confined to a single region as early explanations proposed (He et al., 1998). Rather, motion aftereffects likely depend multiple levels of processing across numerous regions (Mather et al., 2008) and timescales (Shioiri et al., 2021). Thus, targeting component modeling to single regions may miss some of the larger network-wide encoding that may underlie perceptual and cognitive experience.

Related to the targeted, motion-sensitive regions, the motion localizer employed in the current task, while known to produce robust activation in V5/MT+ (Tootell et al., 1995b; Vanduffel et al., 2001; Huk et al., 2002), is a relatively simple form of motion localizer. Other localizers now exist that use more complex motion stimuli (Russ et al., 2021). These methods involve the use of optic flow patterns and structure (Vanduffel et al., 2002; Nelissen et al., 2006) or methods that allow for deciphering the type of motion to which regions respond (Jastorff et al., 2012). The inclusion of such localizers in future work may lead to additional refinement in the characterization of motion-sensitive areas by reducing the potential to overestimate the area of the V5/MT+ complex and thus enhancing the specificity of future results.

### Emotional motion: V5/MT+ activation

Interactions between emotion direction and emotion in the V5/MT+ complex were not observed in the current work. Representational patterns for motion and emotion were found to be orthogonal to each other—they did not manifest as shared representational patterns—suggesting that these features are not interactive in this region. This contrasts previous work showing that changing facial expressions elicits greater activity in V5/MT+ than dynamic neutral faces (Furl et al., 2013). This change, however, may have been driven by the emotionality of the stimuli independent of its motion. As the V5/MT+ ROIs interrogated in the current work (clustered due to their general responsivity to visual motion) are an amalgamation of multiple distinct subregions (Smith et al., 2006; Kolster et al., 2010; Gao et al., 2020), they likely receive signals of motion and emotion from independent sources, modulated by the previous experience with the items. These signals likely includes reciprocal connections with early visual cortices guiding motion perception (Laycock et al., 2007; Vetter et al., 2015), as well as feedback projections from affect-sensitive central structures (e.g., amygdalae) guiding valence representations (Vuilleumier, 2005; Amting et al., 2010; Pessoa and Adolphs, 2010; Mitchell and Greening, 2012; Kryklywy et al., 2020). This suggests that there are multiple channels of processing that occur in the V5/MT+ complex, with potentially distinct circuitry for motion direction and emotion. This is consistent with earlier evidence of anatomical and functional heterogeneity in this region (Smith et al., 2006; Kolster et al., 2010; Gao et al., 2020).

In addition to receiving both emotional and nonemotional visual input, heterogeneity of function for the V5/MT+ complex extends to its visual processing role, independent of emotional signaling. V5/MT+ displays multifaceted encoding of visual signals, including both motion direction (Salzman et al., 1992) and velocity (McKeefry et al., 2008; Grasso et al., 2018), as well as additional roles in motion detection (Newsome and Pare, 1988; Liu and Pack, 2017) and visual prediction (Vetter et al., 2015). The localization of these functional contributions to V5/MT+ versus other visual regions (e.g., area V3; McKeefry et al., 2008) may depend on the familiarity of a visual experience to an observer, rather than the objective visual feature (Liu and Pack, 2017). Subjective changes in visual experience due to changes in V5/MT+ activity may not reflect the objective ability to interact with a moving object (Grasso et al., 2018). In the current paradigm, the use of MAEs to generate perceptual motion may constrain some of the normal visual features used to assess motion direction. For example, by using a consistent velocity and width for visual stimuli in the adaptation period, depth-related cues, such as speed and size (faster/larger at closer distances), may not have been perceptually manipulated in a natural way. Previous work has highlighted these depth features as a driver of activity in V5/MT+ (Nadler et al., 2013; Sanada and DeAngelis, 2014; Kim et al., 2015, 2016), as well as being particularly important for attention allocation (Lin et al., 2008; Rokers et al., 2008). Given the relationship between object depth and approach and avoidance in effective emotional responding (Panksepp, 1990; 1998; Mobbs et al., 2009; Qi et al., 2018; Meyer et al., 2019), additional work is required to fully delineate the impact of depth cues as they relate to emotion representation in regions processing visual motion. Another potentially confounding factor regarding the interpretation of depth and approach cues on object salience is the possibility of physical contact with the object. Approaching cues may be interpreted as on a collision course with the observer, thus gaining additional relevance and influencing V5/MT+ activity in a manner that may supersede the valence-based salience (Lin et al., 2008). This again highlights the need for additional work to fully disentangle the impact of emotion and depth related cues as they pertain to visual processing in V5/MT+.

Current and previous work regarding V5/MT+ emotion-motion integration differed in the way motion direction was presented relative to the observer. Specifically, the present study examined the effects of emotion and direction with stimuli that appeared to change position relative to the observer (i.e., approaching vs receding motion) while previous work often has utilized emotional imagery presented as though viewed by an uninvolved third party (i.e., perpendicular motion). It is possible that emotion has a general priming effect on V5/MT+, wherein increased emotional relevance leads to enhanced motion prediction or preparation of movement regardless of direction. This is consistent with theories that conceptualize threat response as an increase in physiological readiness in times of heightened emotion or stress (Saper, 2002; Tsigos and Chrousos, 2002). Distinct valence-defined representation patterns (POI: IV_Lin_) were identified in V1, ventral visual structures, and V5/MT+ dorsal visual regions. By contrast, univariate analysis conducted in V5/MT+ did not identify valence-based changes in activity (i.e., they showed equivalent activation changes to positive and negative stimuli). Multivariate analyses demonstrated that a sensitivity to nonspecific arousal (POI: IV_All_) was observed with varying strength of expression across all tested visual regions: (i.e., V1, VVS, V5/MT+, *and* DVS). Together, these results suggests that while signals of emotional salience are likely tagged early in visual processing and propagates throughout much of visual cortex, appraisal of the emotional valence direction of these signals is limited to nondorsal regions.

## Conclusions

The current study investigated the impact of emotion and perceived motion on perceptual and neural responses related to visual scenes. Activity in area V5/MT+ was modified by, and showed distinct representational spaces for, both the emotional valence and the perceived motion of an image. Specifically, both approaching and receding images were found to elicit significantly more activity compared with static images, whereas both negative and positive images elicited significantly more activity than neutral images. Furthermore, although similar emotion-related enhancements were observed across widespread areas of the ventral visual stream, no such effects were observed in areas of the dorsal visual stream. Subsequent pattern component modelling performed in visual regions (early, ventral, and dorsal visual regions, as well as V5/MT+) demonstrated again that motion representation in V5/MT+ was similar to that in dorsal visual structures, while emotion in V5/MT+ region was more similar to that in ventral visual structures. Overall, these data provide evidence that visual emotional salience can influence processing in area V5/MT+ and highlights the potential value of motion after-effects for investigating how movement relative to the observer interacts with other stimulus features to affect brain and behaviour.

### Supplementary Information

Below is the link to the electronic supplementary material.

ESM 1 (DOCX 18 kb)

ESM 2 (PNG 123 kb)

High Resolution Image (TIF 149 kb)

ESM 3 (DOCX 19 kb)

ESM 4 (CSV 10 kb)

ESM 5 (CSV 7 kb)

## Data Availability

Raw and partially proccessed data is available for dowload at OpenNeuro.org
