# Supplementary material for: Dissociating representations of affect and motion in visual cortices
Source: Cogn Affect Behav Neurosci. 2023 Aug 1;23(5):1322–45. doi: 10.3758/s13415-023-01115-2 (PMC10545642; doi:10.3758/s13415-023-01115-2)
Supplement: Supplementary file 1 — (DOCX 18 kb) [file 13415_2023_1115_MOESM1_ESM.docx]

**Supplementary Material**

**S1.** Pre-Scan Practice

Participants completed a task designed to induce perceptual motion after-effects on emotionally neutral static images. Specifically, participants viewed a constant pattern of visual motion for a fixed duration before being presented a static neutral image. The pre-scan training consisted of a single testing run lasting ~5 minutes (2 motion direction X 8 target images).

To begin each trial, participants were presented a stationary fixation cross lasting 500ms. Following this, a pattern of constant visual movement (contraction or expansion) was presented for a period of ten seconds. After the completion of the video, a target image appeared immediately, and remained visible for 3000ms. The constant pattern of visual motion induced a pattern of illusory motion in the opposite direction on the subsequent target image. This effect is referred to as the motion-after effect. During the presentation of both the motion pattern and target image, participants maintained fixation on a red centre fixation cross. Once the target image disappeared, two questions were asked in random order. One question required the participant to indicate to what extent the target image appeared to move, while the other required the participant to rate the emotional arousal level of the target image. All responses were given using a 5-point Likert rating, ranging from ‘Very Little’ (1) to ‘Very Much’ (5). All button presses were made with the right hand. The presentation of all motion patterns and target images were randomized within the pre-scan experiment.

**S2.** Bayesian Analyses Reporting

Below we outline extended details for our Bayesian analyses, consistent with the Bayesian Analysis Reporting Guidelines (Kruschke, 2021).

- 1. *Preamble*

We included Bayesian analyses alongside frequentist analyses in order to provide a more thorough picture of results than frequentist analyses alone can offer, especially for null results. All Bayesian analyses that we used aim to express the amount of evidence for the expected pattern of behavioual and neural results given the actual pattern of results.

- 1. *Model description*

For the analysis of the behavioral illusion quality rating and emotional arousal ratings during the fMRI task, separate 3 (MAE Direction: approach, recede, static) X 3 (Emotion: negative, neutral, positive) repeated-measures analyses of variance (ANOVAs) were conducted. All parameters are of direct theoretical interest. These analyses were run for 10,000 iterations, assume a fixed prior distribution of medium effect size for all fixed effects (MAE direction and Emotion), and a random prior distribution of medium to large effect size (“nuisance”) to account for random individual differences.

- 1. *Computation details*

All Bayesian analyses were conducted using the anovaBF function from BayesFactor R package, version 0.9.12-4.4 (Kruschke, 2015). Markov Chain Monte Carlo (MCMC) chains were extracted from posterior distributions for each Bayesian ANOVA using the mcmc.list function coda package, version 0.19-3 (Plummer et al., 2006). We then used the check.psrf function from the zoib package, version 1.5.5 (Liu and Kong, 2015) to calculate all potential scale reduction factors (PSRF) in order to evaluate MCMC chain convergence. All PSRF values for all models were < 1.1, indicating that all MCMC chains successfully converged (Gelman and Rubin, 1992). All PSRF values are reported in supplementary table ST2: PSRF_values.csv.

- 1. *Posterior distribution*

Please see supplemental table ST3: Posterior_stats.csv for medians and 95% credible intervals for all posterior distribution obtained.

- 1. *Decision criteria*

When reporting Bayes factors, we consider any Bayes factor < 3 to be weak or inconclusive evidence against the null hypothesis (Dienes, 2014) across all models. These decisions are theoretically meaningful as a low Bayes factor represents the distribution of data likely being unrelated to the experimental effect of interest.

***References***

Dienes, Z. (2014). Using Bayes to get the most out of non-significant results. Frontiers in Psychology, 5. https://doi.org/10.3389/fpsyg.2014.00781

Gelman, A. and Rubin, D. (1992). Inference from iterative simulation using multiple sequences. Statistical Science, 7(4), 457-511.

Kruschke, J. K. (2015). BayesFactor: Computation of Bayes factors for common designs. R package version 0.9.12-4.4.

Kruschke, J. K. (2021). Bayesian Analysis Reporting Guidelines. Nature Human Behaviour, 5(10), 1282–1291. <https://doi.org/10.1038/s41562-021-01177-7>

Liu, F. and Kong, Y. (2015). ZOIB: an R Package for Bayesian inferences in beta and zero one

inflated beta regression models. The R Journal, 7(2), 34-51.

Plummer, M., Best, N., Cowles, K., & Vines, K. (2019). CODA: Convergence diagnosis and output analysis for MCMC.

**S3:** Whole brain analysis

A 3 (MAE direction) X 3 (emotion) repeated-measures ANOVA was conducted on the whole-brain functional data collected from the main experimental task. All clusters presented in this analysis are significant at *p <* 0.001 (corrected to *p <* 0.01; supplemental table ST2).

Motion after-effect (MAE) direction was found to modulate activity in a single region in right V5/MT+. This was characterized by greater activity for both approaching and receding MAEs compared to the static image condition (*p* < 0.001 for both contrasts), and no differences in activation between MAEs (*p =* 0.17).

Widespread areas were modulated by emotion (see supplemental table ST2). The largest of these clusters was identified within the cuneus and lingual gyrus and characterized by a greater response to negative images compared to positive or neutral images (*p* = 0.003 and *p* > 0.001 respectively) and greater response to positive compared to neutral images (*p <* 0.001). Three additional clusters that transverse areas traditionally identified as contributing to ventral visual processing (i.e., middle temporal, middle occipital and fusiform gyri) were found to greater activity for either emotional image set (positive or negative) compared to neutral imagery (all *p*s < 0.001), but no significant difference between emotional categories (all *p*s > 0.40). A final emotion sensitive region identified within the posterior cingulate cortex was found to have greater activation for negative images compared to positive or neutral images (both *p*s > 0.001 respectively) with no difference in response between positive and neutral images (*p* = 0.90). No MAE direction by emotion interaction was identified in the data
