# Supplementary figures and images for: Dissociating representations of affect and motion in visual cortices
Source: Cogn Affect Behav Neurosci. 2023 Aug 1;23(5):1322–45. doi: 10.3758/s13415-023-01115-2 (PMC10545642; doi:10.3758/s13415-023-01115-2)

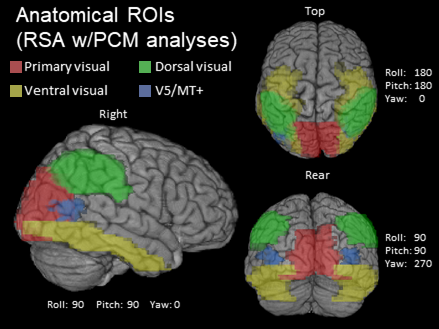

Supplement: Supplementary file 2 — (PNG 123 kb) [file 13415_2023_1115_Fig6_ESM.png]

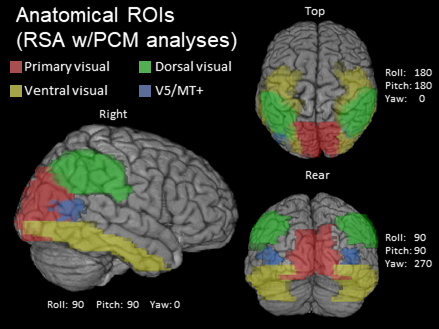

Supplement: Supplementary file 3 — High Resolution Image (TIF 149 kb) [file 13415_2023_1115_MOESM2_ESM.tif]
