# Supplementary material for: Dissociating representations of affect and motion in visual cortices
Source: Cogn Affect Behav Neurosci. 2023 Aug 1;23(5):1322–45. doi: 10.3758/s13415-023-01115-2 (PMC10545642; doi:10.3758/s13415-023-01115-2)
Supplement: Supplementary file 4 — (DOCX 19 kb) [file 13415_2023_1115_MOESM3_ESM.docx]

**Supplementary Table ST1.** IAPS Stimuli

| **Category** | **IAPS Image #** |
| --- | --- |
| Negative |  |
|  | 1052 |
|  | 1220 |
|  | 1275 |
|  | 1280 |
|  | 2055.1 |
|  | 3017 |
|  | 9140 |
|  | 9300 |
|  | 9570 |
|  |  |
| Neutral |  |
|  | 7004 |
|  | 7035 |
|  | 7037 |
|  | 7041 |
|  | 7165 |
|  | 7170 |
|  | 7179 |
|  | 7187 |
|  | 7217 |
|  |  |
| Positive |  |
|  | 1710 |
|  | 2045 |
|  | 2071 |
|  | 2208 |
|  | 2216 |
|  | 2347 |
|  | 7260 |
|  | 7270 |
|  | 7470 |
|  |  |

#### Supplementary Table ST2 Effect of Emotion and Direction during Illusory Motion.

| Effect | Characterization | R/L | Location | BA | X | Y | Z | Vol. (mm^3^) |
| --- | --- | --- | --- | --- | --- | --- | --- | --- |
|  |  |  |  |  |  |  |  |  |
| Direction | (App = Rec) > St | R | MTG | 37 | 43 | -61 | 8 | 480 |
|  |  |  |  |  |  |  |  |  |
|  |  |  |  |  |  |  |  |  |
|  | Neg > Pos > Neu | R/L | Cuneus, LG | 17/18 | 0 | -85 | 4 | 5347 |
|  | (Neg = Pos) > Neu | R | MTG/MOG | 19/37/39 | 45 | 70 | 6 | 2635 |
| Emotion | (Neg = Pos) > Neu | L | MTG/MOG/IOG | 18/19/39 | -44 | -76 | 6 | 2154 |
|  | (Neg = Pos) > Neu | R | FG | 37 | 38 | -47 | -12 | 1466 |
|  | Neg > (Pos = Neu) | L | pCC | 23/30 | 12 | -57 | 10 | 480 |
|  |  |  |  |  |  |  |  |  |

Significant clusters are thresholded at p < 0.001 (corrected to p < 0.05).

App = approaching aftereffects; Rec = receding aftereffects; St = static (null) aftereffects; Neg = negative images; Pos = positive images; Neu = neutral images; MOG = middle occipital gyrus; LG = lingual gyrus; MTG = middle temporal gyrus; PhG = parahippocampal gyrus; pCC = posterior cingulate cortex; PreCG = precentral gyrus.

Note: XYZ are Talairach coordinates and refer to centre of mass.

#### Supplementary Table ST3 Individually defined V5/MT ROIs: Thresholds and Volume

| Participant # | Threshold | Left ROI Vol. (mm^3)^ | Right ROI Vol. (mm^3)^ |
| --- | --- | --- | --- |
|  |  |  |  |
| 1 | *p* < 3*10^-23^ | 636 | 999 |
| 2 | *p* < 4*10^-12^ | 999 | 584 |
| 3 | *p* < 8*10^-45^ | 921 | 999 |
| 4 | *p* < 3*10^-5^ | 986 | 831 |
| 5 | *p* < 7*10^-26^ | 999 | 844 |
| 6 | *p* < 7*10^-25^ | 973 | 441 |
| 7_a_ | *p* < 2*10^-23^ | 752 | N/A |
| 7_b_ | *p* < 1*10^-35^ | N/A | 402 |
| 8 | *p* < 5*10^-15^ | 221 | 999 |
| 9 | *p* < 8*10^-32^ | 363 | 999 |
| 10 | *p* < 6*10^-18^ | 831 | 999 |
| 11 | *p* < 0.002 | 247 | 986 |
| 12 | *p* < 1*10^-15^ | 999 | 506 |
| 13 | *p* < 0* | 260 | 999 |
| 14 | *p* < 1*10^-29^ | 999 | 286 |
| 15 | *p* < 3*10^-12^ | 960 | 999 |
| 16 | *p* < 0* | 727 | 662 |
| 17 | *p* < 2*10^-37^ | 999 | 857 |
| 18 | *p* < 5*10^-14^ | 675 | 999 |
| 19 | *p* < 2*10^-34^ | 986 | 311 |
|  |  |  |  |

*Note:* All thresholds were set to the most liberal threshold which allowed for an identification of bilateral V5/MT+ clusters each > 1000mm^3^. This was not possible for Participant #7 and as such, unique thresholds were used for each hemisphere.

**Supplementary Table ST4:** *Regions of Interest*

| **Region** | **Abbr.** | **Volume (mm^3^)** | **Atlas^1^** | **Label^2^** |
| --- | --- | --- | --- | --- |
|  |  |  |  |  |
| Primary/Secondary visual cortex | *V1/V2* | 77 011 | MNIa_caez_ml_18: | lingual gyrus + calcarine gyrus + cuneus (y > 60) ^3^ |
|  |  |  |  |  |
|  |  |  |  |  |
| Ventral visual structures | *vVS* | 104 730 | MNIa_caez_ml_18: | inferior temporal gyrus + inferior occipital gyrus + fusiform gyrus ^4^ |
|  |  |  |  |  |
|  |  |  |  |  |
| Dorsal visual structures | *dVS* | 79 205 | MNIa_caez_ml_18: | Supramarginal gyrus + Angular gyrus + inferior parietal lobule |
|  |  |  |  |  |
|  |  |  |  |  |
| Visual area 5/MT+ | *V5/MT+* | 11 085 | Localizer scan |  |
|  |  |  |  |  |

^1^ All atlases were transformed to MNI space prior to their implementation or manipulation

^2^ All labels refer to the bilateral structures, unless otherwise indicated

^3^ 37 mm^3^ along the right cuneus were removed from this mask due to overlap with V5/MT+

^3^ 998 mm^3^ along the topmost region of the posterior inferior occipital gyrus were removed from this mask due to overlap with V5/MT+

***See Figure SF1 for visualization of these regions.***
